# β-Hairpin-Based Peptide Hydrogels: The Case of MAX1

**DOI:** 10.3390/gels12020100

**Published:** 2026-01-24

**Authors:** Mariantonietta Pizzella, Valéria Gomes, Enrico Gallo, Sérgio Veloso, Célio Fernandes, Antonella Accardo, Carlo Diaferia

**Affiliations:** 1 IRCCS SYNLAB SDN, Via G. Ferraris 144, 80146 Naples, Italy; mariantonietta.pizzella@synlab.it (M.P.); enrico.gallo@synlab.it (E.G.); 2 Physics Centre of Minho and Porto Universities (CF-UM-UP), Laboratory of Physics for Materials and Emergent Technologies (LaPMET), University of Minho, Campus of Gualtar, 4710-057 Braga, Portugal; 3 Centro de Investigación en Nanomateriais e Biomedicina (CINBIO), Universidad de Vigo, 36310 Vigo, Spain; 4 CEFT—Transport Phenomena Research Center, ALiCE—Associate Laboratory in Chemical Engineering, Department of Mechanical Engineering, Faculty of Engineering, University of Porto, Rua Dr. Roberto Frias, 4200-465 Porto, Portugal; cbpf@fe.up.pt; 5 Department of Pharmacy, Centro Interuniversitario di Ricerca sui Peptidi Bioattivi “Carlo Pedone” (CIRPeB), University of Naples “Federico II”, Via Tommaso de Amicis 95, 80131 Naples, Italy; antonella.accardo@unina.it

**Keywords:** MAX1, peptide hydrogels, β-hairpin peptide, peptide materials, MAX8

## Abstract

This review explores the advancements and applications of β-hairpin peptide hydrogels, starting from the paradigmatic case of MAX1 and its highly versatile analogue MAX8. MAX1 (H-VKVKVKVKV^D^PPTKVKVKVKV-NH_2_) has been identified as the first synthetic β-hairpin peptide for the preparation of stimuli-responsive peptide-based hydrogels. At low ionic strength or neutral pH, MAX1 remains unfolded and soluble. However, under physiological conditions, it folds into a β-hairpin structure, then producing a self-supporting matrix within minutes. The formed gel is shear-thinning and self-healing, making it suitable for injectable therapies. To explore MAX1 molecular space and enhance its practical clinical use, the primary sequence was engineered via chemical modification, with specific single amino acid substitution and relative net charge alteration. This approach generates MAX1 analogues, differing in gelation kinetics, mechanical response and biological performances. The β-hairpin peptide hydrogels are categorized into five different groups: MAX1, MAX1 analogues, MAX8, MAX8 analogues and non-MAX peptides sequences. Collectively, the review outcomes demonstrate the use of β-hairpin peptide matrices as tunable platforms for the development of predictable and stable biomaterials for advanced tissue engineering and drug delivery applications.

## 1. Introduction

In recent decades, there has been a growing interest in the development of innovative self-assembling biomaterials using peptides as fundamental building blocks [1,2,3,4]. Indeed, peptides represent a highly versatile class of molecules due to their biocompatibility, biodegradability, intrinsic self-assembling ability and facile synthesis through solid-phase peptide synthesis (SPPS), allowing precise control over sequence and functionality [5]. Additionally, the inherent ability of peptides to mimic natural protein motifs enables the design of biomaterials that closely resemble the extracellular matrix (ECM), enhancing cell interactions and promoting biological responses [6,7].

Peptide self-organization is driven by non-covalent interactions such as hydrogen bonding, hydrophobic effects, π-π stacking, and electrostatic interactions, which generate secondary structures and supramolecular arrangements. These forces guide the peptides into supramolecular morphologies, including nanotubes [8], nanofibers [9], vesicles [10] and nanostructured films [11]. Among these, peptide-based hydrogels (HGs) have attracted particular attention due to their unique properties [12,13]. HGs are macroscopic, porous, and permeable solids with a biphasic nature (solid/liquid). The solid phase is generally a water insoluble polymer or supramolecular three-dimensional network that, by physically entrapping the liquid, confers non-Newtonian flow features [14]. The nature of cross-links identifies two general categories of hydrogels: chemical and physical ones. In chemical HGs covalent cross-linking bonds generate the network, whereas physical HGs are formed by mutually interacting supramolecular structures via non-covalent interactions.

Peptide-based matrices are formed when monomeric peptides self-assemble into supramolecular elements (e.g., nanofibers or nanotubes) that entail large amounts of water, creating a soft, tissue-like material. Natural and synthetic peptides have been found to be capable of gelling depending on their amino acid sequence, hydrophilic/hydrophobic balance, net charge, and length. Experimental conditions, including peptide concentration, co-solvents, pH, and electrolytes, can be used as gelation triggers, producing responsive systems. This responsiveness opened applicative prospects in the controlled release of therapeutic and diagnostic agents, dynamic changes in mechanical properties, and on-demand gelation, all of which are critical for biomedical applications. Beyond their therapeutic applications, peptide-based hydrogels show considerable potential in diagnostic contexts, as their adjustable physicochemical characteristics and high capacity for biomolecule incorporation support the development of responsive sensing systems, biomarker detection platforms, and imaging-based diagnostic approaches [15,16].

Among peptides, a specific class known as β-hairpin peptides has emerged as particularly effective in forming responsive hydrogels. The β-hairpin (also identified as β-ribbon or β-β element) is one of the structural motifs in proteins. It involves two β-strands, adjacent in primary structure and oriented in an antiparallel direction, linked by a short loop or turn formed by 2–5 residues. β-hairpin motifs are also involved in protein folding, acting as nucleation sites [17], and are important structural domains for protein stability and molecular recognition.

The primary objective of this review is to provide a critical and systematic analysis of the state of the art about design principles, self-assembly mechanisms, and biomedical applications of β-hairpin peptide hydrogels. The β-hairpin peptide hydrogels are categorized into five different groups: MAX1, MAX1 analogues, MAX8, MAX8 analogues and non-MAX peptides sequences.

Starting from MAX1 system, the relationship between primary sequence modifications and the resulting supramolecular morphology and mechanical rigidity is evaluated. The functional versatility of these scaffolds in fields such as drug delivery, antibacterial coatings, and tissue engineering is reported, too. A multi-scale perspective—from molecular folding to macroscopic gelation and applications—was applied.

## 2. MAX1 Peptide

### 2.1. Sequence Identification, Gelation Procedures, and Rheological Characterization

**Figure 1:**
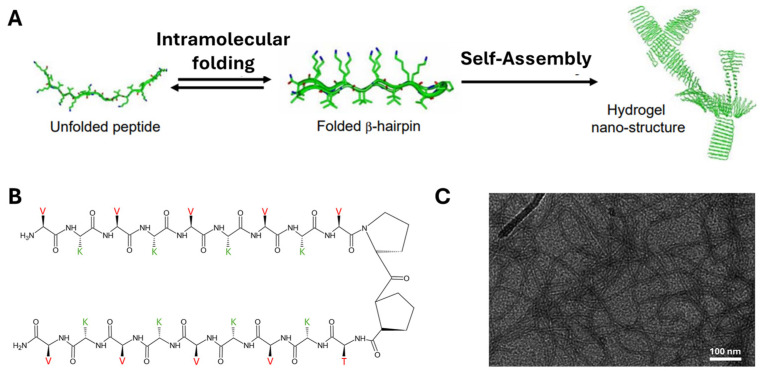
(**A**) Schematic representation of the folding and self-assembly process of MAX1; (**B**) Amino acid sequence and folded β-hairpin conformation of MAX1; (**C**) TEM micrograph (negatively stained with uranyl acetate) showing the fibrillar nanostructures formed upon self-assembly (scale bar = 100 nm). Images adapted with permission from Refs. [21,22].

MAX1 is the acronym of a 20-residue peptide (H-VKVKVKVKV^D^PPTKVKVKVKV-NH_2_), possessing a high propensity for β-sheet organization due to the presence of valine and lysine residues flanking an intermittent tetrapeptide (-V^D^PPT-) designed to adopt type II′ turn structure (see Figure 1) [18]. A Type II β-turn is a common four-residue secondary structure motif in proteins (residues *i* to *i* + 3) that causes a reversal in the direction of the polypeptide chain. It is stabilized by a hydrogen bond between the carbonyl oxygen of residue *i* and the amide hydrogen of residue *i* + 3 [19].

The type II′ β-turn is also known as a “mirror-image” turn or a diastereomer of the more common type II β-turn. Due to this diastereotopic relationship, type II′ and type II turns have identical ϕ and ψ angles but with opposing signs [20].

The β-hairpin (with β referring to the dihedral angle that defines the peptide secondary structure) formation in the self-assembled state is promoted by arranging the polar lysine and apolar valine residues flanking the β-turn in an alternating manner.

MAX1 was de novo designed and synthesized for the first time by Schneider et al. in 2002 [18]. It was found to be capable of forming responsive hydrogels via a pure, fully reversible self-assembly mechanism, driven by both the intramolecular folding of β-hairpin peptides and their propensity to self-assemble.

Under basic aqueous solution conditions, where some of the lysine side chains of MAX1 are deprotonated, this peptide intramolecularly folds into an amphiphilic β-hairpin where one face of the hairpin is lined with hydrophobic valine residues and the other face is lined with hydrophilic lysine residues. After intramolecular folding, subsequent self-assembly of monomeric hairpins is facilitated both laterally via H-bond formation between distinct hairpins and facially through hydrophobic association of the valine-rich faces. Importantly, intramolecular folding is designed to be reversible. The reversibility of the self-assembly process was investigated by gradual acidification of the solution below the intrinsic pK_a_ of the lysine side chains (~10.7), resulting in intrastrand charge repulsion between neighbouring lysine residues and subsequent unfolding of individual hairpins, ultimately controlling the self-assembled hydrogel structure.

Gelation was achieved by a 1/1 *v*/*v* dilution step of MAX1 (dissolved in water or in 125 mM borate/10 mM NaCl, pH = 9.0) into buffer (250 mM borate and 20 mM NaCl, pH = 9.0), followed by gentle pipet mixing [18]. The MAX1 critical gelation concentration (CGC) was found to be 1.0 wt%, and gel kinetics required several hours to be complete. Rheological studies corroborated both the gelation pathway and CGC. At 1.0 wt%, a crossover liquid-like to gel-like transition behaviour was detected, with G′ (elastic or storage modulus) > G″ (viscous or loss modulus) [18]. An increase in G′ was observed increasing the concentration to 2.0 wt% (G′ = 1600 Pa), and a Newtonian behaviour was verified at pH = 6.0, thus suggesting the complete supramolecular gel disassembly. Similarly, to other HGs, MAX1 exhibits a shear-thinning behaviour, with a decrease in viscosity as strain rate increases, and self-healing properties, with a rapid recovery after shear stress stops. Interestingly, after the application of a high strain (1000% strain), the MAX1 matrix quickly reforms, recovering 80% of its original G′ modulus in 30 min. These entire rheological tests suggest a relatively rigid and fast-recovering profile for MAX1 gels [18]. The remarkable properties of the MAX1 hydrogel have made it appealing in the field of materials science. In the following years, further studies explored its characteristics in depth, also evaluating its responsiveness and the conditions that favour the formation of stiffer hydrogels.

Building on their earlier work, the same authors explored the role of ionic strength in regulating hydrogel formation [23]. They discovered that in a salt-free environment at physiological pH, MAX1 remains unfolded, while upon increasing the ionic strength of the solution, electrostatic interactions between Lys residues are screened, and a β-hairpin conformation is adopted. Moreover, it was observed that higher salt concentrations promote the formation of stiffer hydrogels. For instance, for HGs formed at 2.0 wt% peptide concentrations, the use of a 400 mM NaCl solution generated matrices with a G′ of 3000 Pa, whereas in lower salt conditions (20 mM NaCl) a softer material (G′ = 100 Pa) was formed. Salt concentration also influences the kinetics of self-assembly and network formation. This trend was confirmed by rheological experiments, in which an increase in the NaCl concentration caused a rapid increase in G′ (within few minutes) due to the self-assembly process. Conversely, relatively slow rates were observed for solutions with lower salt concentration. These results clearly indicated that salt concentration could be used as a parameter to fine-tune hydrogel stiffness and gelation kinetics. Additionally, temperature was also shown to influence gelation, with faster assembly occurring at 37 °C compared to 20 °C.

Based on this evidence, one of the most common media for in vitro cell testing, DMEM (Dulbecco’s Modified Eagle’s Medium), was tested as a solvent for inducing the MAX1 gelification [21]. The procedure involved dissolving MAX1 in sterile water and adding an equal volume of serum-free DMEM to achieve a final peptide concentration of 2.0 wt%. Rheological studies indicated that under these conditions, MAX1 rapidly forms stiff, self-supporting hydrogels (G′ = 1500 Pa in 30 min, increasing to 2500 Pa in 2 h). Importantly, the hydrogel remained stable and further increased its mechanical profile (G′ = 10,000 Pa) under cell culture conditions, highlighting its robustness in biological environments. Additionally, fibroblast cells (NIH 3T3) adhered to and proliferated on the matrix surface, confirming MAX1 cytocompatibility and reinforcing its potential as a scaffold for cell-based therapies.

In this case as well, the self-assembly of MAX1 into a fibrillar hydrogel network was found to be a temperature-dependent process. Subsequently, MAX1 hydrogels were reported to undergo a stiffening transition as they are cooled below a critical temperature (21 °C), but only when boric acid is used to buffer the initial peptide solution [24]. Rheological studies carried out on a MAX1 solution (0.5 wt%, pH = 9.1 with 125 mM borate) highlighted an increase in G′ (from ∼100 to ∼600 Pa) upon heating in the range between 5 and 40 °C. The storage modulus remained stable over time (up to 2 h). Cooling the gel (from 40 to 5 °C) caused an additional G′ increase to 2000 Pa (stiffening transition at ∼21 °C). It can be supposed that this increase in G′ is related to temperature-dependent changes in peptide solubility. This stiffening was found to be a fully reversible process, as the stiffness of the hydrogel decreased when the temperature increased back to 40 °C. Interestingly, the inclusion of glucose in the gel formulation disrupts this temperature-related stiffening behaviour. The gel still forms, but glucose competes with the peptide for borate binding, and at high enough concentrations (25–50 mM), this prevents the borate–peptide complexation that is responsible for stiffening.

**Figure 2:**
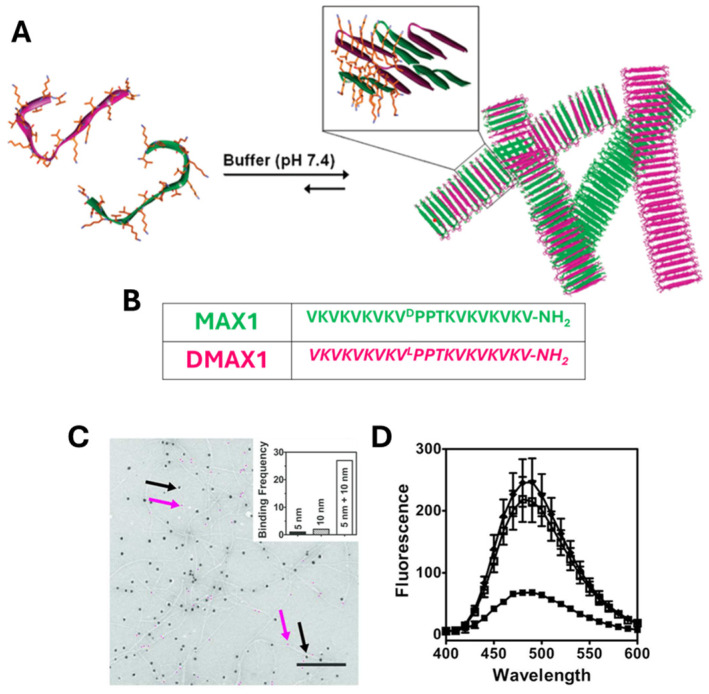
(**A**) Assembly mechanisms of enantiomeric peptides leading to the formation of a fibrillar network. Enantiomers can either self-sort, generating uniform fibrils (solid-coloured fibrils), or co-assemble, producing a network composed of fibrils containing both enantiomers (multi-coloured fibrils). (**B**) Amino acid sequences of the enantiomers MAX1 and DMAX1 reported in one-letter code. Co-assembly of MAX1 and DMAX1 enantiomers is demonstrated by nanoparticle labelling of peptide fibrils using TEM (**C**) and by fluorescence-quenching assays (scale bar = 200 nm) (**D**). Images adapted with permission from Refs. [25,26].

To further enhance the mechanical properties of MAX1, Nagy and coworkers formulated the hydrogel by mixing MAX1 with its *D*-enantiomer (see Figure 2A,B) in a 1/1 molar ratio [25]. The gel formation was induced by adding an equal volume of gelation buffer to the aqueous solution of the racemic mixture, yielding a final concentration of 1.0 wt% (50 mM BIS-TRIS propane, 150 mM NaCl, pH 7.4). Under these experimental conditions, the racemic mixture generated gels more rapidly and with a fourfold increase in mechanical rigidity (800 Pa) with the single-enantiomer hydrogels. It was also demonstrated that the specific chiral relationship between MAX1 and *D*MAX1 was crucial for the enhancement of rigidity, as rheology experiments using a mixture containing a control peptide did not show a significant increase in material rigidity.

The physical basis for this enhancement remained unclear until 2017, when Nagy-Smith et al., using various microscopy and modelling approaches, revealed the unique MAX1/*D*MAX1 co-assembly, which increases both cross-linking density and fibril stiffness [26]. In their study, the authors used transmission electron microscopy (TEM), to demonstrate that the two peptides form a co-assembled network. In particular, they examined the ability of MAX1-Azide and *D*MAX1-Biotin derivatives to bind gold nanoparticles (GNPs) with a size of 10 and 5 nm, respectively. TEM images showed fibrils decorated with both 5 and 10 nm GNPs, indicating co-assembly (see Figure 2C). On the other hand, for fluorescence microscopy experiments, MAX1-EDANS (fluorophore) and DMAX1-Dabcyl (quencher) peptides were used to detect proximity within fibrils (see Figure 2D).

Mixed fibrils showed strongly reduced fluorescence due to the efficient quenching occurring at very short distance (<3 nm) between EDANS and Dabcyl, confirming the co-assembly of peptides. The increased rigidity reported for MAX1/*D*MAX1 HGs was not attributed to a smaller mesh size within the gel, but rather to a higher bending modulus of the fibrils, as revealed by Small Angle Neutron Scattering (SANS) and Diffusing Wave Spectroscopy (DWS). Molecular-level analysis suggested that the enantiomers alternate within each fibril monolayer, forming a rippled-sheet structure. This arrangement promotes tight packing of hydrophobic valine side chains, which stabilizes the fibrils and increases their stiffness, an organization not achievable in enantiomerically pure fibrils. The study also showed that altering side chain interactions can further tune the gel’s mechanical properties. These findings indicate that chirality can be used as a design principle to control the mechanical behaviour of self-assembling materials.

### 2.2. Structural Basis of MAX1 Hydrogel Formation and Mechanical Behaviour

Over the years, not only the macroscopic features of the MAX1 hydrogel and the optimal conditions to improve its stiffness have been elucidated, but several studies have also highlighted key structural insights that have improved the understanding of its gelation, self-assembly and mechanical properties. Structural and computational studies have enabled the optimization of the design of MAX1 hydrogel for advanced biomedical applications, including tissue engineering and drug delivery. The first insight into the MAX1 fibrillar network was reported in 2004, revealing that it could serve as a synthetic peptide model for cytoskeletal networks [22]. The folded β-hairpin peptides form fibrils with a well-defined cross-section at the nanoscale, exhibiting rheological properties similar to those of semiflexible biopolymers with permanent cross-link points, such as F-actin and DNA. Using TEM imaging, small-angle neutron scattering (SANS) and rheology, the authors revealed that MAX1 assembles into uniform nanofibrils (~3 nm in diameter, extending hundreds of nanometers in length) behaving as rigid rods, with a cross-sectional diameter of approximately 30.9 Å. Rheological measurements demonstrated that the hydrogels exhibit semiflexible network elasticity, with the G′ significantly greater than the G″ across a frequency range of 0.1–10 rad/s, indicating mechanically stable networks. The G′ scaled with peptide concentration as G′ ∝ c^2.5^, and for 6.1 mM peptide, G′ reached 1600 Pa, corresponding to a network mesh length of approximately 55 nm. The results suggest that the self-assembled peptide fibrils form permanent cross-links, distinct from entangled networks. A deeper insight into the dynamics of hydrogel formation was achieved by applying microrheology and circular dichroism (CD) spectroscopy to track the gelation process in real time [27]. Fluorescent polystyrene (PS) microspheres were used as probe particles to monitor MAX1 gelation through the mean squared displacement (MSD), revealing that the gel formation occurs approximately 9 min after initiation for hydrogels at pH 9 with 125 mM borate and 10 mM NaCl. The G′ ranged from 0.3 to 2.4 Pa for concentrations of 0.056 to 0.15 wt%, in agreement with bulk rheology. Moreover, the gelation time decreased with increasing concentration, from 30 min at 0.05 wt% to 11 min at 0.15 wt%. The study also demonstrated that β-sheet formation, monitored by CD spectroscopy, directly correlated with hydrogelation, with a threshold CD signal at 216 nm (−10,000 to −12,000 deg·dmol^−1^·cm^2^) required for gelation. These insights helped refine hydrogel processing conditions for biomedical applications.

Two distinct kinetics events occurring on different time scales were detected during the MAX1 hydrogelation process (at pH = 7.0, 50 mM bis-TRIS propane buffer, 0.4 M NaCl): early-stage fibril cluster formation, followed by intercluster overlap and clusters percolation, with a critical gelation point identified at approximately 56 min [28]. Rheological analysis confirmed that mechanical properties evolved over time. The study also compared MAX1 self-assembly mechanism to other biopolymeric networks, highlighting its distinct nanostructural features, such as monodisperse fibril dimensions and unique branching mechanisms. These findings provide valuable insights into peptide self-assembly kinetics, offering strategies for homogeneous cell encapsulation in hydrogels for tissue regeneration by appropriately timing cell introduction during gel formation.

The shear-thinning and self-healing properties of MAX1 hydrogel were further investigated, revealing that it exhibited immediate recovery post-shear, though initial G′ value was lower than pre-shear level [29]. For MAX1, increasing shear duration at 1000 s^−1^ (5, 40, 120 s) resulted in progressively lower initial G′ values (440 Pa, 180 Pa, 86 Pa), recovering to 95%, 76%, and 65% of the original modulus (2900 Pa) after two hours. Scattering analyses (SANS and SAXS) showed no fibril alignment under shear, confirming that shear-thinning occurs through domain fracture rather than fibril breakage. The cylindrical fibril structure remained intact, with a radius of ~16 Å. The self-healing model suggests gel domains percolate upon shear cessation, with higher pre-shear stiffness leading to stronger post-shear recovery.

To understand the structural features that contribute to the network’s morphology and mechanical rigidity of the MAX1 hydrogel, Miller et al. investigated the relative arrangements of individual hairpins within the fibrils using computational modelling techniques [30]. The study suggested the presence of polymorphic hairpins arrangements within the fibrils. However, the relative populations and conformational energies of these polymorphic arrangements indicated a preference for an arrangement in which the hairpin turn regions are unable to form intermolecular interactions. Repulsive intramolecular electrostatic interactions appear to dictate the formation of fibrils with shorter persistent lengths. These repulsive forces also disfavour fibril entanglements. Taken together, the modelling predicts that MAX1 forms a network containing many branch points, a network morphology supported by the formation of short fibril segments. Moreover, under static conditions, the preferred branched structures of the MAX1 peptide assembly result in a cross-linked hydrogel organization. At the same time, the shear stress leads to short fibrillar structures, thus to a more fluidic like hydrogel states.

In the same year, another study conducted by Nagy-Smith and colleagues used experimental techniques instead of computational modelling to directly observe the fibril structure [31]. Using solid-state NMR, MAX1 was found to adopt a β-hairpin conformation, forming assemblies of monomorphic, double-layered cross-β architectures. Unlike polymorphic amyloid fibrils, MAX1 fibrils align in a *Syn*-orientation within β-sheets and in an *Anti*-orientation between stacked layers, stabilized by electrostatic and hydrophobic interactions. Gelation occurs within one minute as β-sheet-rich nanofibrils assemble into a stable, interpenetrating 3D network. Solid-state NMR, TEM, and AFM confirmed the uniform fibrillar morphology. MAX1 fibrils contain a hydrophobic core and solvent-exposed lysine residues that prevent uncontrolled aggregation. Their structural homogeneity stands in contrast to the variability typically observed in amyloid fibrils, demonstrating that β-hairpin-based peptide designs can yield predictable, stable biomaterials for functional applications.

To further investigate the assembly process, Adams and colleagues used vibrational spectroscopy with carbon–deuterium (C–D) labelling, providing high spatial and temporal resolution [32]. Time-resolved stopped-flow FTIR (SF-FTIR) experiments revealed that MAX1 self-assembly follows a single exponential pattern, with significant changes in the central region of the β-hairpin. Temperature-induced gelation triggered a transition from an unfolded to a folded state, corresponding to a shift from a hydrophilic to a hydrophobic environment. Distinct absorption shifts were observed for terminal, central, and turn valine residues. Terminal residues (Val1 and Val20) experienced moderate shifts, whereas central residues (Val5 and Val16) displayed larger shifts and narrower absorption lines, indicative of tighter packing within the hydrophobic core. On the other hand, turn residue Val9 exhibited an intermediate shift. The study also compared enantiopure and racemic MAX1 gels, finding that racemic gels were stiffer due to the formation of heterochiral fibrils. SF-FTIR data were complemented by CD and rheology, collectively highlighting the cooperative nature of the assembly process. This combined approach provides a detailed characterization of self-assembly kinetics and hydrophobic core formation, showing that site-specific SF-FTIR is a powerful method for studying peptide assembly.

### 2.3. Functional Versatility of MAX1 Hydrogels in Biomedical Applications

Expanding the functional scope of MAX1, Salick and colleagues demonstrated that MAX1 hydrogel surfaces exhibit antibacterial properties, making them valuable for wound healing and implant coatings [33]. Co-culture studies showed that MAX1 hydrogel (2.0 wt%) effectively inhibited both Gram-negative (*E. coli*) and Gram-positive (*Klebsiella pneumoniae*) bacteria, while still promoting fibroblast proliferation. Notably, hemolysis tests confirmed that MAX1 is non-toxic to red blood cells, reinforcing its biocompatibility and clinical potential. Subsequently, it was also demonstrated that MAX1 hydrogels are able to encapsulate and controllably release model biomacromolecules [34]. Fluorescence recovery after photobleaching (FRAP) measurements of diffusion coefficients for FITC-labelled dextrans within gels of varying weight percentages showed that probe diffusion depends on probe size, peptide sequence, and gel mesh size. Bulk release studies from 0.5 wt% and 2.0 wt% gels confirmed that release rates align with FRAP-measured diffusion trends. The complete release of dextran probes and lactoferrin ranged from days to over a month, indicating that hydrogel mesh size and electrostatic interactions influence release rates.

Moreover, mesh size can be tuned by adjusting peptide weight percent or sequence, with higher weight percent gels being more rigid and having smaller mesh sizes. It is known that MAX1 HG does not provoke pro-inflammatory response in vitro [35], as also demonstrated by the minimal TNF-α secreted by macrophages cultured on the hydrogel.

**Figure 3:**
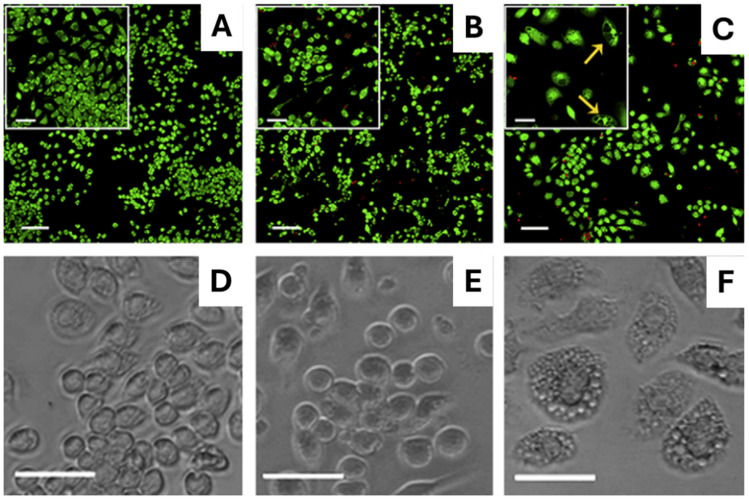
Cellular viability and morphology of J774 macrophages after 48 h cultured on: (**A**,**D**) TCTP control; (**B**,**E**) 2.0 wt% MAX1 hydrogel; and (**C**,**F**) TCTP control þ 5 mg/mL LPS. Top panels (**A**–**C**): LSCM images showing live/dead assays, scale bar = 100 μm. Bottom panels (**D**–**F**): phase-contrast images illustrating cellular morphology, scale bar = 50 μm. Images adapted with permission from Ref. [35].

These findings were supported by live/dead assays and phase-contrast microscopy, which showed that macrophages attached and proliferated on the hydrogel without signs of activation or cytotoxicity. Additionally, in control experiments, macrophages activated with lipopolysaccharide (LPS) showed typical signs of inflammation (see Figure 3).

## 3. MAX1 Peptide Analogues

To overcome the limitations of the original MAX1 peptide and expand its functionality, a wide array of analogues has been developed through rational design. These modified β-hairpin peptide analogues retain the fundamental self-assembly mechanism of MAX1 but incorporate strategic sequence alterations to modulate their folding behaviour, gelation kinetics, mechanical properties, environmental responsiveness, and biological compatibility. These analogues not only provide insights into the structure–function relationships governing peptide self-assembly but also enable the development of hydrogels with tailored properties suited for various biomedical applications. In the following sections, these MAX1-derived peptides have been categorized in (i) hydrophobicity tuning, (ii) turn region substitutions, (iii) electrostatic alterations, (iv) stimuli-responsive behaviour, and (v) biofunctionality. This subclassification, which follows the nature and purpose of their modifications, has been introduced to facilitate a structured and comparative discussion of their design principles and material properties.

### 3.1. Hydrophobicity Modification for Thermal Gelation Control

It is well established that hydrophobic interactions can play a crucial role in peptide self-assembly and gelation. In this context, specific **MAX1** residues were strategically modified to investigate the effect of the different hydrophobicity on both the gelation temperature (T_gel_) and kinetics (see peptide sequences reported in Table 1). Historically, the first analogues reported in the literature were **MAX2** and **MAX3**, in which only Val7 or both Val7 and Val16 are replaced with the less hydrophobic threonine residue [36]. Results indicate that the progressive decrease in hydrophobicity causes an increase in the T_gel_ (∼25, ∼40 and ∼60 °C for MAX1, MAX2, and MAX3) for hydrogels prepared under the same experimental conditions (2.0 wt% in 125 mM Borate, 10 mM NaCl, pH 9). In contrast to MAX1 and MAX2, MAX3 exhibits fully reversible folding and gelation behaviour upon temperature cycling (from an unfolded structure at 5 °C to a β-sheet structure at 80 °C at a concentration of 150 µM). The reversibility was also demonstrated by rheology with reversion from a self-supporting hydrogel (G′ ~1100 Pa) at 75 °C to a low-viscosity solution (G′ ~0) at 5 °C. This reversibility is explained by the weaker hydrophobic interactions in MAX3 which allow rapid unfolding upon cooling, while in MAX1 and MAX2, increased hydrophobicity stabilizes the assembled state more strongly, making the unfolding and dissolution kinetically slow or inaccessible. In the same study, a control peptide with a ^D^Pro^L^Pro-to-^L^Pro^L^Pro mutation in the turn region fails to fold into a β-hairpin and instead forms irreversible fibrillar aggregates, confirming that β-hairpin formation is essential for hydrogelation.

Building on this, two additional analogues, named **MAX4** and **MAX5**, were developed to further dissect the role of lateral hydrophobic interactions and the β-turn composition [37]. In MAX4, the serine in the β-turn is replaced by a threonine residue, and the positions of valine and lysine residues are reversed respect to MAX1 whereas in MAX5 only the Ser-to-Thr substitution is present. The Val-Lys inversion in MAX4 is aimed at understanding the impact of Val-Val lateral interactions on the hydrogelation process. Structural characterization highlighted that even though MAX4 folds into β-sheet structures at similar temperatures to MAX1 (T_gel_ ~26–27 °C), its assembly kinetics are significantly slower, reaching equilibrium in ~250 s at 40 °C compared with 100 s for MAX1. Moreover, MAX4 forms much weaker hydrogels at 1 wt% (G′ ≈ 350 Pa), and TEM images reveal a heterogeneous structure comprising single fibrils and higher-order tapes, in contrast to the uniform fibrils of MAX1. These results highlight the importance of lateral valine packing in dictating assembly kinetics and hydrogel architecture. MAX5, by contrast, assembles nearly identical kinetics and rigidity to MAX1 (G′ ≈ 1000 Pa at 1 wt%), and its morphology is also indistinguishable. This confirms that the reduced rigidity and altered morphology in MAX4 arise specifically from disruption of lateral hydrophobic interactions rather than from turn-region modifications.

**Figure 4:**
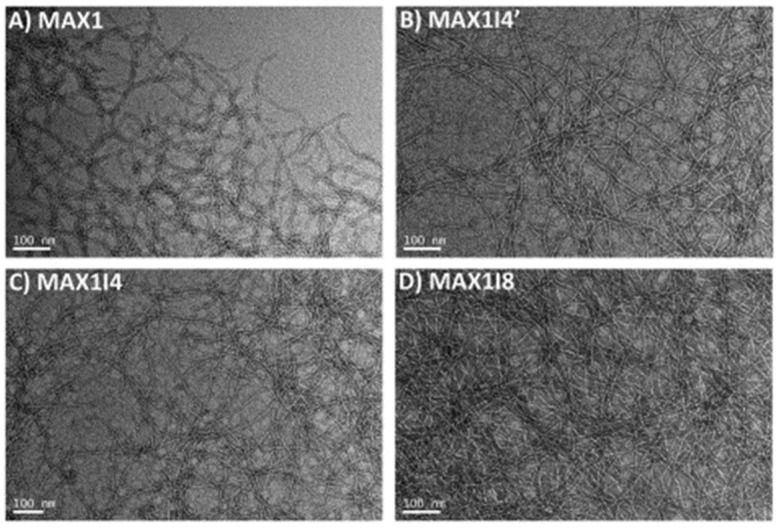
TEM images of 2.0 wt% peptide hydrogels showing fibril morphology. Scale bars = 100 nm. Images adapted with permission from Ref. [38].

To investigate the effect of increasing hydrophobicity, new analogues (**MAX1I4**, **MAX1I4′**, and **MAX1I8**) were designed by substituting valine with isoleucine, which is known for its higher hydrophobicity and stronger β-sheet forming propensity, thereby incorporating four or eight isoleucine residues at different positions [38]. These analogues retain MAX1’s β-hairpin self-assembly mechanism but exhibit distinct gelation behaviours depending on the number and placement of isoleucine residues. For these peptides, the hydrogel was prepared by initially dissolving the peptide at 4 wt% in 25 mM HEPES buffer (pH 7.4), followed by mixing with DMEM medium (1:1 ratio) to reach a final concentration of 2 wt%. MAX1I8 formed a self-supporting gel within 5 min, followed by MAX1I4 (15 min), MAX1I4 (30 min), and MAX1 (~1.5 h), demonstrating the impact of isoleucine content on gelation kinetics. Rheological measurements showed that the time required to reach G′ = 100 Pa was less than 30 s for MAX1I8, 10 min for MAX1I4, 30 min for MAX1I4′, and ~75 min for MAX1. After 4 h, G′ values were 492 Pa (MAX1I4), 942 Pa (MAX1I4′), and 1494 Pa (MAX1I8). MAX1I8 also exhibited shear-thinning behaviour and rapid recovery (G′ ~1640 Pa within 30 min following application of 1000% strain), suggesting its suitability for injectable applications. CD measurements confirmed β-sheet formation upon DMEM addition, and TEM showed ~3 nm fibrils for all peptides (Figure 4). Moreover, MAX1I8 demonstrated excellent biocompatibility, supporting robust NIH-3T3 fibroblast proliferation (MTT absorbance ~2.0 at day 5) and low immune activation (IL8 and TNFα levels ~100 fold lower than lipopolysaccharide controls). Its rapid gelation kinetics also facilitated uniform cell encapsulation, mitigating sedimentation issues observed with slower gelling systems such as MAX1.

Another series of analogues of MAX1 was designed in which all eight valines were replaced with other hydrophobic residues, including linear aliphatic amino acids such as aminobutyric acid (**M(Abu)**), norvaline (**M(Nva)**), and norleucine (**M(Nle)**) as well as phenylalanine (**M(Phe)**) and isoleucine (**M(Ile)**) [39]. CD experiments showed that all peptides could fold into β-sheet structures upon thermal triggering, with folding midpoint temperatures (T_g_) decreasing with increasing hydrophobicity: Tg_M(Abu)_ > Tg_M(Nva)_ > Tg_M(Nle)_ (70, 30 and 5 °C for M(Abu), M(Nva) and M(Nle), respectively). pH-dependent CD analyses also confirmed that more hydrophobic peptides folded at lower pH values, indicating that hydrophobicity, rather than β-sheet propensity, served as the primary driver of folding. Folding and gelation of 1 wt% peptide solutions at pH 9 were induced by increasing the temperature to 80 °C for M(Abu) and 50 °C for the others. Rheological results (G′ ~3300 Pa and ~680 Pa for M(Nva) and M(Nle), respectively) revealed the absence of a direct correlation between gel stiffness and hydrophobicity or β-sheet propensity, suggesting that material rigidity depends on side chain packing and network branching. TEM and SANS confirmed that all peptides formed well-defined fibrils of ~3.2–3.5 nm in diameter. However, higher-order fibril organization was residue-dependent: M(Nle) and M(Phe) formed ropes and braids, while MAX1 and M(Ile) remained as single fibrils.

### 3.2. Charge and Electrostatic Interaction Control

As hydrophobicity also the electrostatic interactions, including net charge and responsiveness to the ionic environment, can significantly affect peptide assembly and hydrogels properties under physiological and ionic stimuli. In this context, different MAX1 analogues, reported in Table 2, have been designed with varied charges or ion-binding capabilities. In these studies, MAX1 folding is restricted to high pH due to electrostatic repulsion arising from its eight lysine residues (net charge of +9). To modulate gelation conditions, a series of MAX1 analogues were designed through site-specific substitutions of lysine with glutamic acid, thereby tuning the net charge and electrostatic profile of the peptides [40]. These variants fold and assemble under milder, more physiological conditions, depending on their net charge and the specific site of substitution. One notable analogue, **MAX1(K15E)** (net charge of +7), displayed enhanced folding kinetics due to reduced intramolecular electrostatic repulsion and formed hydrogels at 0.5 wt% (pH 7.4, 150 mM NaCl, and 37 °C), conditions under which MAX1 barely gelled. Moreover, rheological measures revealed that the hydrogel formed by MAX1(K15E) exhibits significantly higher stiffness (G′ ~600 Pa) compared to MAX1 (G′ ~50 Pa). Variants with intermediate charges (e.g., **MAX1(K15I)** or **MAX1(K15T)**, net charge of +8) showed correspondingly intermediate gelation rates and stiffness, confirming a correlation between net charge, assembly kinetics, and mechanical properties. Importantly, peptides with excessive charge reduction (e.g., **MAX1(K4E, K15E)**, net charge of +5) precipitated instead of forming gels, indicating that a balance between electrostatic screening and hydrophobic collapse is critical. TEM images revealed similar fibril morphology (~3 nm in width) across all variants, suggesting that local structure remains conserved despite differences in gelation behaviour.

Another MAX1 analogue, **MARG1**, has been developed to serve as an injectable, antibacterial biomaterial with activity against methicillin-resistant *Staphylococcus aureus* (MRSA) [41]. As previously noted, the MAX1 hydrogel exhibits antibacterial properties against drug-susceptible strains but lacks efficacy against resistant ones [33]. To enhance its antibacterial performance, MARG1 incorporates two arginine residues (at positions 6 and 17) into its 20-residue sequence, in addition to six lysine ones. The guanidinium groups of Arg are known to interact strongly with negatively charged bacterial surface molecules, potentially improving antimicrobial activity. In aqueous solution, MARG1 remains unfolded due to electrostatic repulsion. Upon addition of DMEM, which contains mono- and divalent salts, these charges are screened, inducing folding into an amphiphilic β-hairpin. This structure self-assembles into β-sheet-rich fibrils, resulting in the formation of a mechanically rigid hydrogel. The gelation process can be carried out directly within a syringe, enabling injectability. Rheological characterization demonstrates that at 2 wt% the MARG1 hydrogel forms rapidly at 37 °C, reaching a G′ of 2200 Pa within 60 min. The hydrogel exhibits shear-thinning and self-healing properties, flowing under high strain (1000%) and recovering ~50% of its stiffness within seconds after the removal of stress. Biological assays indicate that MARG1 outperforms MAX1 in eradicating MRSA, particularly at high bacterial densities (up to 2 × 10^8^ CFU/dm^2^). While both hydrogels inhibit methicillin-susceptible *S. aureus* (MSSA), only MARG1 retains full activity against MRSA. The antibacterial effect requires direct contact with bacteria, likely via membrane disruption, and is not mediated by peptide diffusion. Importantly, MARG1 is non-cytotoxic to mammalian stem cells, demonstrating its biocompatibility.

A rationally designed analogue of MAX1 (named *Peptide 1*) was developed to investigate the role of intermolecular metal coordination—specifically Zn^2+^ binding—in promoting peptide self-assembly and hydrogelation [42]. In contrast to MAX1, *Peptide 1* incorporates four His residues in place of Lys near the termini and substitutes Val residues with the more hydrophobic Leu ones to enhance the mechanical properties of the resulting hydrogel. This design aimed to enable gelation at physiological pH by reducing charge repulsion and introducing potential Zn^2+^ coordination sites. *Peptide 1* was shown to form hydrogels at a concentration of 1.0 wt% in 50 mM buffer over the pH range 7.0–9.0 and in the presence of 10 mM ZnCl_2_, MgCl_2_, CaCl_2_, or 30 mM NaCl at pH 7.4. Notably, Zn^2+^ promoted stronger β-sheet formation and appeared to participate in intermolecular coordination. Rheological analysis at 37 °C demonstrated formation of moderately stiff hydrogels with a G′ of ~170 Pa (ZnCl_2_) and ~180 Pa (NaCl), values comparable to those previously reported for MAX1 [18], while the G″ remained low (~6 Pa), indicating predominantly elastic behaviour. Importantly, *Peptide 1* hydrogels exhibited shear-thinning behaviour, rapidly recovering G′ and G″ following large deformations (γ = 1000%). TEM imaging revealed fibrillar networks with uniform diameters (~3.2–3.8 nm), consistent with β-hairpin stacking.

In order to produce β-hairpins with acidic hydrophilic faces, some analogues were designed to incorporate negatively charged residues such as aspartic acid (Asp), glutamic acid (Glu), and aminoadipic acid (Aad) (see Table 2) [43]. This modification enables the formation of hydrogels with a net negative charge, intended to enhance compatibility with specific cell types whose phenotypes are sensitive to the electrostatic properties of their environment. In this case, β-hairpin folding is induced by protonation of acidic side chains and/or by temperature-driven hydrophobic collapse. CD analyses confirmed that folding is both pH- and temperature-dependent: VD1, containing Asp, folded only under acidic conditions (pH < 5); VE1, containing Glu, folded at pH < 7; and VX1, containing Aad, folded at pH ≤ 8. Oscillatory rheology showed that at 0.5 wt% VD1 formed hydrogel only at pH 4.6, reaching a G′ of ~1500 Pa after 60 min at 50 °C. VE1 and VX1 exhibited similar behaviour, with VX1 forming stiffer, faster-gelling networks at lower pH values. However, none of the first-generation peptides formed gels under physiological conditions. To overcome this limitation, second-generation designs (**VE3** and **VEQ1**) introduced strategic charge-reducing substitutions: VE3 by replacing two Glu residues with Lys, and VEQ1 by introducing uncharged glutamine residues. Both VE3 and VEQ1 self-assembled into β-sheet-rich fibrillar networks at pH 7.4 and 37 °C, forming hydrogels within seconds. Time-sweep rheology at 0.5 wt% demonstrated rapid gelation with final G′ values of 1386 ± 162 and 1463 ± 91 Pa for VE3 and VEQ1, respectively. These hydrogels also exhibited shear-thinning with full or near-complete recovery post-shear, making them suitable for syringe-based delivery. Cell encapsulation experiments using C3H10T1/2 cells demonstrated excellent cytocompatibility for both VE3 and VEQ1 hydrogels. Despite the transient exposure to basic pH during gelation, nearly all cells remained viable after 5 h, and confocal microscopy confirmed a uniform distribution of cells throughout the gel matrix. These results validate the suitability of these anionic hydrogels for 3D cell culture and injectable therapeutic delivery applications.

**Figure 5:**
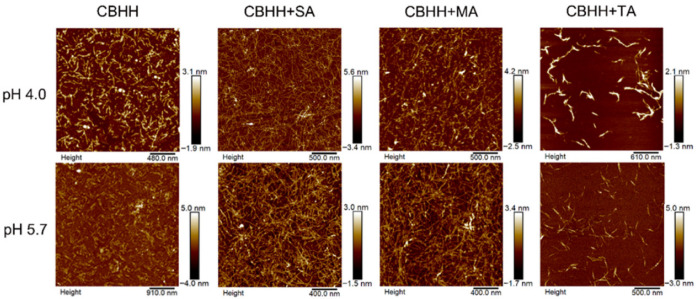
Atomic force microscopy (AFM) images of CBHH (2.0 mM) and CBHH with dicarboxylates at a 1:1 molar ratio, recorded at pH 4.0 and 5.7. Images adapted with permission from Ref. [44].

A further analogue, named **CBHH,** was then developed to enable hydrogel formation through co-assembly with dicarboxylate sodium salts under mildly acidic conditions [44]. Unlike MAX1, CBHH contains His residues that confer pH-sensitive folding and electrostatic interaction with dicarboxylates, offering a tunable platform for self-assembly. The objective was to promote β-sheet formation and nanofiber gelation via electrostatic attraction and hydrogen bonding, modulated by the presence of hydroxylated dicarboxylates: succinic acid (SA), malic acid (MA), and tartaric acid (TA). CD spectroscopy confirmed that all three dicarboxylates facilitated a conformational transition of CBHH from random coil to β-sheet at pH 4.0 and 5.7. AFM imaging revealed that co-assembly with SA and MA induced the formation of longer, thinner nanofibers, which are entangled into hydrogel networks, whereas TA increased the fibre width via lateral aggregation without forming a stable gel (Figure 5). Rheological analysis at 4.0 mM CBHH and dicarboxylate (1:1 molar ratio, pH 5.7) demonstrated the formation of self-supporting hydrogels for CBHH/SA and CBHH/MA, with G′ of ~350 Pa and ~470 Pa, respectively. In contrast, CBHH/TA exhibited a lower G′ (~100 Pa) and remained a viscous solution. At pH 7.0, all systems showed enhanced mechanical strength and complete recovery following shear thinning, indicating the formation of robust and reversible hydrogel networks. Cell viability assays using NIH-3T3 fibroblasts showed ~50% survival on CBHH alone, which increased to ~80% upon the addition of dicarboxylates, likely due to the charge neutralization reducing cytotoxic effect. These results suggest that dicarboxylate-assisted co-assembly of CBHH yields tunable, biocompatible hydrogels with promising applications for tissue engineering applications. Finally, the analogue **H4LMAX** was developed by substituting Val residues with Leu and replacing four of the Lys residues with His, thereby reducing the net charge to +5 and enabling spontaneous gelation at physiological pH [45]. Moreover, to enhance the interaction of the gel with cells, two H4LMAX analogues (**H4LMAX-RGDS** and **H2LRDMAX)** were also synthesized. In H4LMAX-RGDS the original peptide was derivatized at its C-terminus with the well-known RGDS sequence, whereas in H2LRDMAX a pseudo-RGD motif was inserted by replacing two His residues with dipeptide Arg-Asp. All peptides formed transparent hydrogels at 1 wt% in 50 mM TRIS buffer (pH 7.4). Rheological measurements at 37 °C (0.2% strain, 6 rad/s) showed G′ of 40 Pa for H4LMAX-RGDS, 210 Pa for H4LMAX, and 510 Pa for H2LRDMAX. Although all the peptides supported osteoblast viability, H4LMAX-RGDS was found to be able to promote superior cell morphology and was selected for subsequent lactoferrin (LF) delivery studies.

Hydrogels loaded with 80 µg of LF retained ~40% of the protein after 5 days, despite electrostatic repulsions, likely due to histidine-mediated interactions. The hypothesis was that the mesh size of H4LMAX-RGDS (51.7 ± 1.8 nm) permitted LF diffusion, and TEM images confirmed partial LF binding to peptide fibrils. LF-loaded hydrogels enhanced osteoblast viability (*p* < 0.05), confirming the osteoinductive potential of the system. In conclusion, these results suggest that H4LMAX-RGDS hydrogel combines injectability, cytocompatibility, integrin-mediated cell adhesion, and sustained delivery of bioactive LF, making it a promising candidate for bone tissue engineering applications.

### 3.3. Activation by External Stimuli: Light, pH, and Mechanical Stress

**Figure 6:**
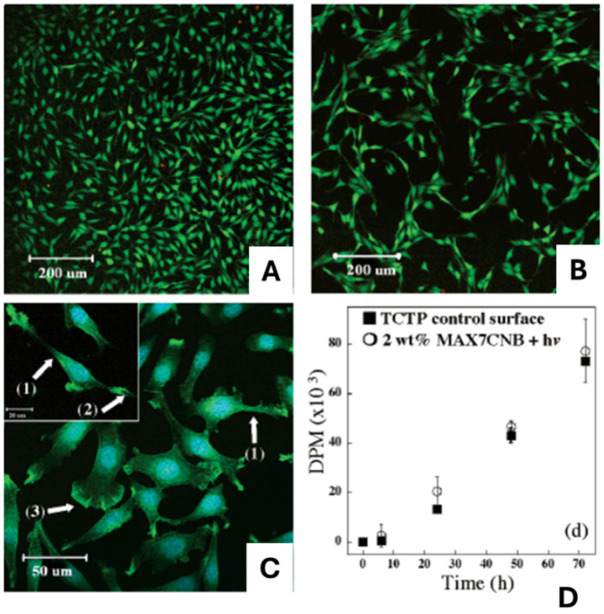
(**A**) Live/dead assay of NIH3T3 fibroblasts on decaged MAX7CNB hydrogel after 24 h (green = live, red = dead; LSCM) (scale bar = 200 µm). (**B**) Same assay on sterile borosilicate glass (control) (scale bar = 200 µm). (**C**) LSCM at 8 h: actin stress fibres (green) highlight lamellipodia (1), filopodia (2), and ruffled membrane of a migrating cell (3); nuclei stained blue (scale bar = 50 µm). (**D**) Proliferation rates measured via [^3^H]thymidine uptake on 2.0 wt% MAX7CNB gel versus tissue culture polystyrene (control). Image adapted with permission from Ref. [46].

In this section and in Table 3 are summarized all the responsive MAX1 analogues, which, by reacting to external triggers such as light, pH changes, or mechanical forces, offer dynamic control over peptide folding and material properties, enabling applications that require spatiotemporal precision and adaptability. Among these analogues, **MAX6**, **MAX7**, and **MAX7CNB** have been developed to form light-triggered, cytocompatible hydrogels without the need for macromolecular precursors or photoinitiators [46]. These analogues exploit the precise control provided by light to initiate peptide folding and subsequent self-assembly into hydrogels, an approach well-suited for biomedical and microfluidic applications. The difference between these analogues lies in the amino acid in position 16. MAX6 contains a Glu, which, due to its negative charge, can disrupt the peptide folding under physiological conditions; MAX7 contains a Cys residue, which supports both folding and potential disulfide dimerization; and finally, MAX7CNB features a Cys modified with a 2-nitrobenzyl photocage that prevents the folding until UV exposure. As expected, CD spectrum of MAX6 (150 µM, pH 9.0) showed a typical dichroic signature of unfolded peptides. In contrast, MAX7 folds into β-hairpins under the same conditions, while MAX7CNB remains unfolded under the ambient light due to the charged photocage but undergoes rapid folding and hydrogelation upon UV exposure (λ > 300 nm). At 2.0 wt%, MAX7 forms rigid hydrogels, while MAX7CNB initially behaves as a low-viscosity solution, but transforms into a rigid hydrogel following UV irradiation, with G′ of ~1100 Pa and ~1000 Pa, respectively, indicating strong, elastic networks. G″ values were over an order of magnitude lower than G′, confirming the formation of solid-like, elastic networks. Approximately 60% of MAX7 peptides form disulfide-linked dimers. MAX7CNB hydrogels support NIH-3T3 fibroblast adhesion, spreading, migration, and proliferation, demonstrating good surface cytocompatibility (Figure 6). Cells exhibited defined actin structures and typical morphology. These findings confirm that β-hairpin folding and self-assembly into hydrogels can be precisely controlled via light, enabling the formation of cytocompatible, mechanically tunable hydrogels under mild conditions using de novo designed peptides. Such systems hold promises for applications in tissue engineering, microfabrication, and responsive biomaterials.

Successively, a pH-responsive cryogel based on the peptide derivative **VK20** was described which due to the presence of a methacrylate can be covalently grafted onto a polyethylene glycol (PEG)-based polymer network via UV-initiated cryopolymerization. This system integrates molecular conformational changes with macroscopic shape transformations, thus enabling a multifunctional, reprogrammable shape memory effect (SME) [47]. In the resulting cryogel, the microporous matrix stabilizes shape and dimensional integrity while preserving reversible peptide folding. The microporous architecture of the hydrogel facilitates compressibility through pore wall buckling, enhances ion diffusion for rapid pH responsiveness, and minimizes volumetric swelling. At slightly acidic pH (~6.5), peptides adopt a random coil conformation, rendering the hydrogel soft and deformable. Upon increasing pH to ~9.5, the peptide fold into β-sheet structures, leading to additional physical cross-linking that stiffened the hydrogel and fixed the deformed shape. Cryogels were synthesized by copolymerizing PEGdiCEMA with VK20 peptides, to form an interpenetrating polymer network. Shape memory cycling involved deformation at pH 6.5, fixing shape at pH 9.5, and recovering original shape upon returning to pH 6.5, with complete recovery observed within 2 h. Rheological measurements showed a marked increase in G′ as a function of the pH (from ~0.1 kPa at pH 6.5 to ~1 kPa at pH 9.5). These results were consistent with FTIR and NMR data indicating ~33% reversible peptide conformational switching. Swelling decreased by 13–35% with pH increase, reflecting volume stability imparted by the porous structure. Shape fixity ratios ranged from 38 ± 5% to 90 ± 3%, increasing with peptide content, while shape recovery ratios reached 90–98%. Recovery kinetics were slower at higher peptide concentrations, likely due to delayed disassembly of β-sheet cross-links. Control experiments confirmed that the SME originated from peptide folding rather than from simple protonation effects. This system demonstrates hierarchical integration of molecular conformational switching and macroscopic responsiveness, advancing the development of pH-triggered shape memory hydrogels for biomedical applications.

A tyrosine-functionalized analogue was developed to enable post-assembly covalent cross-linking, thereby enhancing mechanical rigidity [48]. Tyr residues in the peptide undergo selective oxidation by potassium nitrosodisulfonate (Frémy’s salt), a long-lived radical known to convert the phenolic ring of Tyr into reactive o-quinone intermediates. This chemical strategy offers advantages over enzymatic oxidation (e.g., via tyrosinase), particularly in pre-formed gels where mesh size may restrict enzyme diffusion.

**Figure 7:**
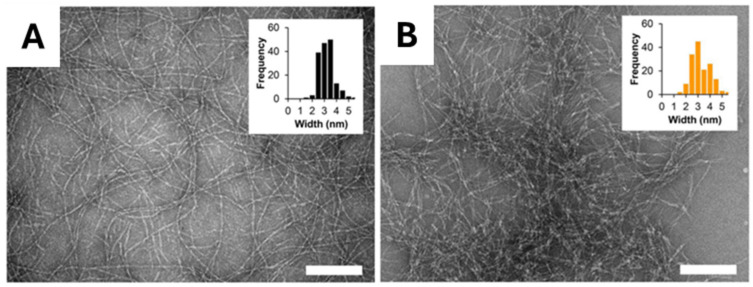
TEM images of fibrils extracted from 1.0 wt% fibrillar gel networks: (**A**) without oxidant treatment and (**B**) after overnight oxidation using Frémy’s salt. Scale bar = 100 nm. Fibril widths were measured using ImageJ software, with sample sizes of n = 164 for the non-oxidized gel and n = 156 for the oxidized gel. Image adapted from Ref. [48].

Upon addition of 300 µL of 100 mM Frémy’s salt to pre-assembled hydrogels (1.0 wt% peptide in HEPES buffer), oxidation was complete following overnight incubation, as evidenced by a visible colour change and UV–vis spectral shifts (appearance of peaks at 300 and 475 nm). TEM revealed that while fibril morphology (~3 nm in width) remained intact, cross-linked networks showed increased fibrillar entanglement (Figure 7). Rheological measures demonstrated that oxidized gels exhibited an ~8-fold increase in storage modulus (G′ = 25,470 ± 6723 Pa) relative to unoxidized controls (G′ = 2932 ± 401 Pa). Shear-thinning experiments confirmed the injectability of the gel, with oxidized gels partially recovering (~60%) their rigidity post-shear, whereas unoxidized gels fully recovered (105 ± 3%). The mechanical enhancement was dependent on the Frémy’s salt concentration; homogeneous cross-linking and significant increases in stiffness were observed only at oxidant-to-tyrosine ratios of ≥33:1. Cytocompatibility assays using human dermal fibroblasts indicated high cell viability on both oxidized and control gels, provided residual oxidant was thoroughly removed. Furthermore, cross-linked gels exhibited enhanced resistance to enzymatic degradation by trypsin after 48 h. This post-assembly cross-linking strategy thus represents a robust and tunable approach for increasing the stiffness of peptide hydrogels while preserving their structural integrity and biocompatibility, making them suitable for applications in tissue engineering and regenerative medicine.

### 3.4. Structural Modifications for Morphological Control

This category encompasses analogues specifically designed to modulate the supramolecular architecture of the hydrogel network (see Table 4). Rather than altering assembly kinetics or responsiveness to external stimuli, these modifications aim to influence fibril morphology, packing interactions, and the overall structural organization of the gel. Strategies include the introduction of steric constraints, modulation of backbone flexibility, or incorporation of bulky side chains (e.g., glycans) to tune fibril bundling and branching behaviour.

A notable example of this class of analogues is **LNK1**, which was engineered to limit branching during nanofibril growth through steric specificity within the assembled fibril’s hydrophobic core [49]. In this design, the non-turn Val residues of MAX1 were substituted by 2-naphthylalanine (Nal) and Ala residues, with significantly larger and smaller side chain volumes, respectively. This asymmetric side chain architecture promotes specific, shape-complementary packing, thereby reducing structural defects such as fibril branching, which is commonly observed in MAX1 assemblies. Both peptides are folded into β-hairpin structures and self-assemble into nanofibrils with comparable morphology (∼3 nm diameter) under physiological conditions (0.5% *w*/*v* peptide, pH 9, 125 mM boric acid, 10 mM NaCl, 37 °C). However, their network architecture and mechanical responses differ markedly. Contrarily to MAX1, LNK1 hydrogels, although initially robust (G′ ≈ 200 Pa), failed to recover post-shear, with G′ decreasing to ~5 Pa, indicating irreversible network disruption. Molecular dynamics (MD) simulation studies confirmed enhanced rigidity, reduced β-sheet twist, and minimized internal fluctuations in LNK1 fibrils. The lock-and-key hydrophobic interface of LNK1 effectively suppresses fibrils branching and enhances structural order, albeit at the expense of shear responsiveness. To further investigate structural control via post-translational-like modifications, another series of analogues (**P1–P5**) were designed incorporating enhanced aromatic sequons (EASs), natural N-glycosylation motifs capable of to stabilizing protein β-turns [50]. The goal was to explore how EAS-mediated glycosylation influences peptide assembly, fibril network formation, and hydrogel mechanics. Unlike MAX1, which contains a synthetic β-turn, P1–P5 analogues, and their glycosylated counterparts **GP1–GP5**, incorporate native sequons (FANGT or YNGT) into β-hairpin-forming peptides and display N-acetyl-glucosamine (GlcNAc) moieties within either hydrophilic or hydrophobic environments. All peptides assembled under mildly promoting conditions (pH 7.4, 37 °C, 1.0 wt%) to sensitively probe the impact of glycosylation. While glycosylation generally had minimal effect on fibril morphology or width, it significantly influenced fibril network evolution and the mechanical properties of the resulting gels. For instance, GP1 exhibited delayed network formation and formed a substantially softer gel compared to P1 (G′ = 284 ± 59 Pa vs. 824 ± 51 Pa). Similarly, GP2, with the glycan positioned on the hydrophobic face, displayed an 85% reduction in G′ compared to P2 (18 ± 7 Pa versus 109 ± 18 Pa). GP3 also formed a weaker gel than P3 (364 versus 637 Pa) and exhibited delayed network maturation (Δt = 47 min vs. 10 min). In contrast, glycosylation had minimal impact on systems with a high intrinsic propensity for assembly, such as P4, P5, GP4 and GP5, which incorporate both the FANGT turn and a Glu-Lys salt bridge. These peptides formed hydrogels with comparable G′ values under standard conditions. Overall, glycosylation was found to retard gelation kinetics and reduce gel stiffness; however, these effects can be mitigated by promoting strong peptide assembly. These findings offer valuable design principles for engineering glycopeptide-based biomaterials with tunable mechanical properties.

## 4. MAX8 Peptide

**Figure 8:**
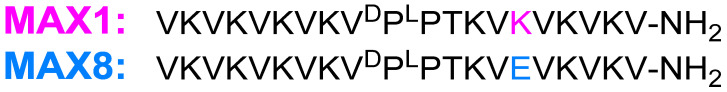
Peptide sequences of MAX1 and MAX8, according to the one letter code.

To optimize mesenchymal cell encapsulation, Schneider’s group designed a 20-amino acid residue peptide by simply—but decisively—replacing the lysine residue at position 15 in the MAX1 peptide sequence with a glutamic acid residue (Figure 8) [51]. This resulted in **MAX8**, which is structurally similar to the parent while possessing a lower overall charge. The rationale was to decrease the amount of positive charge to be screened, making self-assembly faster and more efficient using the same gelation conditions; when in β-hairpin state, the three-dimensional structure is stabilized by salt bridges between the glutamic acid and the cross-strand Lys residue. The shorter gelation kinetics of MAX8 allowed homogeneous cell incorporation since gelation occurs before cell deposition.

As previously pointed out in this study the authors further confirmed that rational design, especially modulating peptide charge, can be used to control the rate of secondary structure folding and subsequent gelation without changing the local morphology of the hydrogel fibrils [51]. For example, self-assembly kinetics may not have a direct impact on the on-site structure but can influence the morphology at larger length scales This suggests that it is possible to address current (bio)technological problems by selecting the physical, chemical, and structural properties of materials.

In the following chapters, we attempt to relate the molecular design and rheological behaviour of the β-hairpin-forming peptide MAX8, highlighting its high mechanical versatility and biocompatibility, and understand how these properties promote or limit its applications.

### 4.1. Linking Structure, Mechanics, and Function in MAX8 Hydrogels

Once again, SANS remains a key tool in evaluating the three-dimensional matrix formed by MAX8 at scales ranging from a few angstroms to a fraction of a micrometre. Unlike other techniques, such as TEM, this approach allows the gels’ architecture to be probed in their swollen state, uncovering key aspects for understanding mechanical, swelling, and transport properties. Low scattering angles, referred to as the low-*q* regime, provide information about large-scale heterogeneities and network domains, while high scattering angles, at high *q*-values, reveal information about cross-link density and network size.

**Figure 9:**
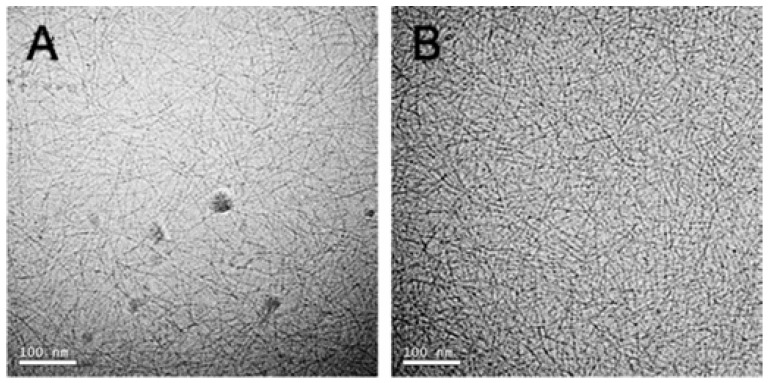
Cryo-TEM images of 0.5 wt% (**A**) and 2.0 wt% (**B**) MAX8, showing the increase in hydrogel network density (scale bars = 100 nm). Retrieved from Ref. [52].

Using this technique, Hule and co-workers [52] sought to understand the relationship between the structure and properties of hydrogels formed by the new MAX8 peptide. The authors found that low-*q* scattering exponents do not vary over hundreds of nanometres, regardless of MAX8 peptide concentration, indicating a similar micro-scale morphology [52]. However, the scenario at the nanoscale proved to be different: the high-*q* scattering exponent increases with increasing MAX8 concentration, which, together with the decrease in correlation length (a parameter taken from the fitting of SANS results that is related to the interfibre distance), points to an increase in physical cross-link areas, as shown in Figure 9.

The following year, the same authors investigated the relationship between peptide sequence, structure, and hydrogels properties formed by MAX1 and MAX8 peptides. They evaluated how replacing the Lys residue in MAX1 with Glu in MAX8 affects the dynamics of fibres at nanoscale and, whether these differences could explain the observed disparities in the bulk mechanical behaviour of both hydrogels [53]. NSE (Neutron Spin Echo) experiments revealed that both gels follow the semiflexible chain model, indicating that fibres are neither completely flexible nor rigid [53]. By fitting mathematical models to the NSE data, Branco et al. [53] deduced the segmental diffusion coefficient, a measure of how fast an individual fibre moves due to thermal fluctuations, and found that fibres in MAX1 are more mobile than those in MAX8. Knowing the viscosity of the medium and the bending modulus for both peptides, the authors determined the lower cutoff length (the length below which a fibre is rigid and above which it is flexible): 3.0 ± 0.2 nm for MAX1 gel and 3.6 ± 0.2 nm for MAX8 hydrogel. This corresponds to the number of hairpin units along a fibre that are inflexible due to strong electrostatic interactions. Considering a hairpin width of 0.5 nm [46], the hairpins are packed in groups of 6 in MAX1 and 7 in MAX8. This difference is consistent with the presence of a salt bridge in MAX8 (Glu-Lys) that strengthens intermolecular interactions, increasing fibre rigidity and lowering the segmental diffusion coefficient. However, these differences in fibre packing are too small to explain the much higher elasticity of MAX8 compared to MAX1: 2500 Pa versus 880 Pa for 1.5 wt% and 1700 Pa versus 200 Pa for 1.0 wt%, respectively. Here, the number of physical cross-links plays an important role. Low-*q* SANS data (probing larger scales) show that MAX8 gels are less homogeneous, indicating clusters of densely cross-linked areas; this is the key factor that explains not only the considerably higher elasticity of the MAX8 gel compared to MAX1 [53] but also the increase in elasticity with concentration [52]. These regions are likely to result from the faster self-assembly of the MAX8 peptide, as this phenomenon leads to the formation of fractal imperfections, increasing the number of fibre branches [52,54].

Such studies paved the way for the rational design of peptide hydrogels that, under the same conditions (temperature, pH, salt and peptide concentration), exhibit fundamentally different structural properties. Initially, it was thought that rapid self-assembly and gelation were direct consequences of favourable β-hairpin folding. However, further investigation revealed that the situation is more complex: folding is highly stabilized by the self-assembly process. Indeed, what was previously thought to be the cause (efficient β-hairpin folding) is the effect of the self-assembly process; and the supposed effect (self-assembly and gelation) is, in fact, the driving factor that promotes and maintains the folded state. This reverse causality highlights the dynamic nature of these systems, where structure and assembly are more interdependent than linearly ordered processes.

**Figure 10:**
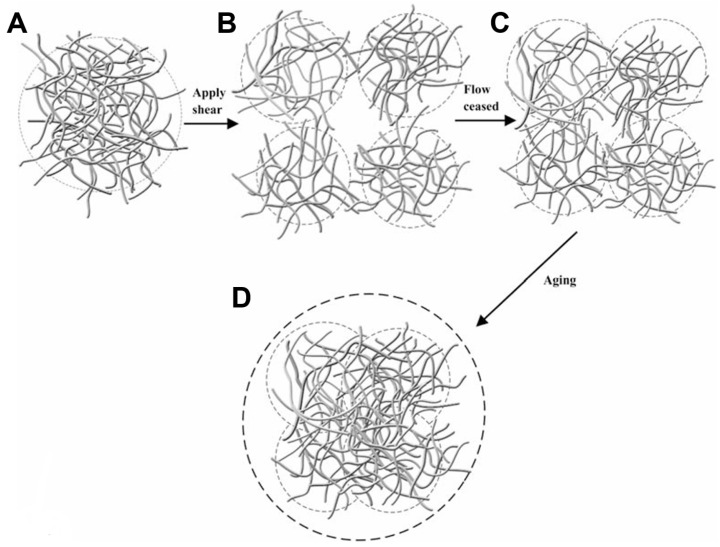
(**A**–**D**) Yan et al. proposed mechanism underlying the recovery of the gel-like state of β-hairpin gels after the application of shear stress. Adapted with permission from Ref. [29].

With an improved understanding of how peptide sequence and network morphology affect the bulk mechanical behaviour of this β-hairpin gel, it becomes pertinent to investigate its shear-thinning and recovery capacities. In a first experiment, MAX8 hydrogels were individually subjected to a constant shear rate over varying time periods to assess the impact of shear duration on recovery kinetics [29]. Although the gels recovered their solid-like state under all conditions, the percentage of elasticity recovered 2 h after cessation of the shear was dependent on the duration of the applied stress: 89%, 80% and 77% for 5, 40 and 120 s of shear, respectively. In a second experiment, the authors subjected an equivalent set of hydrogels to different shear rates for the same time duration. Similarly, all gels recovered a portion of their elasticity with percentages depending on the shear rate: 98%, 86% and 80% for shear rates of 10 s^−1^, 100 s^−1^ and 1000 s^−1^, respectively. In order to simulate the shear associated with syringe injection, the MAX8 hydrogel was injected into the rheometer through a needle, and its recovery into a solid gel was subsequently observed. Furthermore, Yan et al. [29] evaluated both the effect of peptide concentration and ionic strength on the recovery capacity of the gels. Here, they found that, for stiffer hydrogels (resulting from higher peptide concentration or higher ionic strength), both the “immediately post-shear” G′ and the “2 h-post-shear” G′ are larger than in (initially) softer gels. Nevertheless, all hydrogels showed similar percentages of elasticity recovery, indicating the same mechanism of shear-thinning and healing. In addition to these observations, it was observed that during flow, there is no breakdown of the gel network into individual fibres nor alignment of the latter, with the 1D NSE fittings showing a fibrillar radius of approximately 16 Å (consistent with previous calculations [52,53]). By combining these findings, the authors proposed the mechanism underlying shear-flow and immediate recovery of β-hairpin gels, as illustrated in Figure 10 [29]. This mechanism is based on the existence of a hydrogel whose 3D-network is formed by physical cross-links of fibres’ branches and entanglements (Figure 10A). The application of shear flow breaks the network into domains that allow the gel to transition to a fluid state enough to flow through a needle (Figure 10B); once the mechanical stress stops, these domains immediately percolate into each other, forming a network that extends throughout the gel, which now exhibits a solid-like mechanical response (Figure 10C). Finally, over time, the gel’s rigidity is restored through deeper penetration of the fibres from different domains (Figure 10D). This mechanism explains why the recovery of β-hairpin gels is affected by the intensity and duration of shear flow, as mentioned earlier. Such stress can cause small fragments of the network to detach from the domains and become trapped in the pores, thus no longer contributing to the rigidity of the hydrogel. Consequently, with increasing duration and shear rates, more fragments are formed, leading to lower recovered elasticity, consistent with the rheological data. It also explains why initially stiffer hydrogels present higher final G′ values than weaker ones. This occurs because stiffer networks have a higher cross-link density (in agreement with studies [53,54]) and so do their domains during flow; in fact, a matrix that reforms from more cross-linked domains is naturally stiffer throughout the recovery process.

Based on their physical, chemical and structural advantages, MAX8 hydrogels were immediately explored, similarly to their MAX1 counterparts, for biomedical applications. The first step in moving towards the clinic was to assess their biocompatibility. This was done by evaluating the in vitro immune response (reflected by the secretion of pro-inflammatory cytokine TNF-α) of MAX8-exposed mouse macrophages [35]. It was found that neither bulk- nor thin film-hydrogels induced cytokine release, as observed when cells are stimulated with bacterial lipopolysaccharide. Microscopy confirmed the non-cytotoxicity of MAX8 gels, and that they support the normal morphology of macrophages. Hence, their biocompatibility combined with fast gelation, mechanical strength and injectability under physiological conditions make them highly attractive for tissue engineering and both drug and cell delivery. Being highly tunable according to preparation conditions, these platforms have the potential to bridge the gap between supramolecular peptide assemblies and medical translation, as shown in the following chapters.

#### 4.1.1. Cell Culture, Encapsulation and Delivery

**Figure 11:**
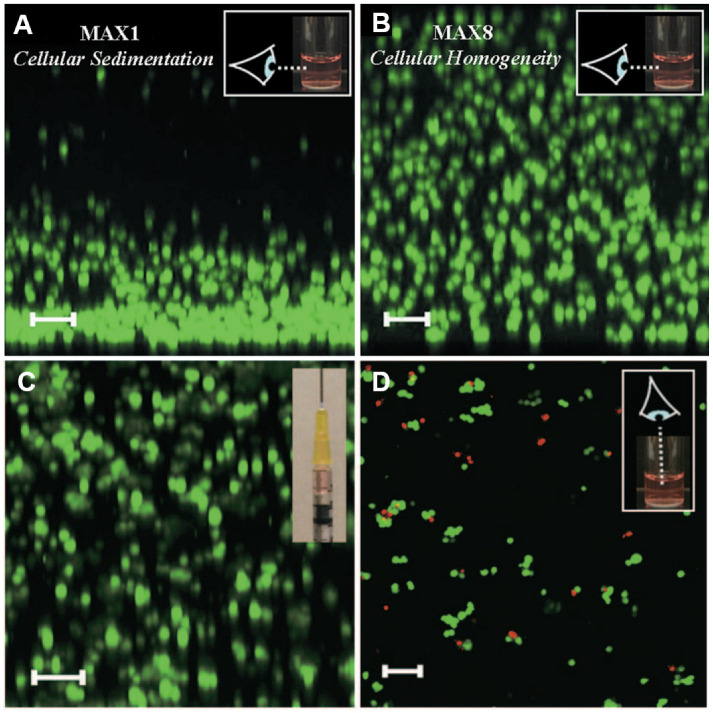
LSCM z-stack images (along the *y*-axis) of C3H10t1/2 stem cells encapsulated in 0.5 wt% MAX1 (**A**) and MAX8 (**B**) hydrogels (cells labelled with Cell Tracker Green). LSCM z-stack image (along the *y*-axis) of C3H10t1/2 stem cells encapsulated in 0.5 wt% MAX8 hydrogel, shear-thinned by syringe (**C**) (cells labelled with Cell Tracker green). (**D**) LSCM z-stack image (along the *z*-axis) of Live-Dead assay of C3H10t1/2 stem cells 3 h after syringe shear-thinning (scale bars = 100 μm). Adapted from Ref. [51].

Upon its conception, MAX8 hydrogels were immediately tested for cell encapsulation and delivery. They fulfilled two fundamental requirements for this purpose: (i) rapid gelation in order to homogeneously incorporate cells into the network (overcoming the limitations of MAX1 gels) and (ii) the ability to remain localized at the site of introduction (compared to less viscous hydrogels or those applied in the sol state) [51]. Moreover, in order to deliver gel/cell constructs via syringe, hydrogels must also be self-healing and recover quickly after shear; indeed, Haines-Butterick et al. [51] found that MAX8 gel recovery kinetics are even faster than the initial gelation kinetics, anticipating what was later confirmed in [29]. Furthermore, recovery kinetics proved to be comparable in the presence of cells, which are viable, evenly distributed throughout the matrix, and remain at the application site even when disturbed. Figure 11 shows the comparative distribution of C3H10t1/2 cells in MAX1 and MAX8 hydrogels and, in the latter, before and after syringe-induced shear-thinning.

In another study, a capillary with a “clinically relevant geometry” (inner diameter of 250 µm) was used to mimic the injection of MAX8 hydrogel with a 26-gauge needle and thus study how it flows during this process [55]. A plug-flow profile was found: a central region where the gel and cells experience almost no shear and a narrow region near the capillary walls that actually senses shear and where network deformation takes place. This time, MG63 osteoblasts were encapsulated in MAX8 hydrogels, and once again, Live/Dead assay showed minimal loss of cell viability and uniform distribution after injection. In addition, it was found that the hydrogel’s stiffness (controlled by peptide concentration) influences the plug size and, in turn, shear protection: the higher the G′, the wider the plug region, providing greater shielding for the cells. This study is of great importance from a biotechnological point of view, as it shows that hydrogel-encapsulated cells (in this particular case) are better preserved than buffer-dispersed ones, during injection [55].

#### 4.1.2. Drug Delivery

MAX8 hydrogel has also proven advantageous in the field of drug delivery. After finding that it had a protective effect on cells [55] and given the problem of medium to long-term instability of some proteins, Lindsey envisaged this platform as a controlled delivery system for nerve growth factor (NGF) and brain-derived neurotrophic factor (BDNF)—clinical options for treating spinal cord injuries [56]. Experiments showed that the release of MAX8 hydrogel-encapsulated proteins depended directly on the initial cargo loading and inversely on MAX8 peptide concentration (the higher the peptide concentration, the greater the number of cross-links, resulting into a smaller mesh size [53]). Biologically, the released growth factors remained active and functioning normally for longer periods (even after 28 days) and, in response, the tested cells (PC12) prolonged neurite-like processes, with better results compared to using in-solution NGF/BDNF. Hence, MAX8 hydrogel proved to effectively protect fragile proteins such as NGF and BDNF, enabling sustained long-term release controlled by the concentration of therapeutic proteins and hydrogel-forming peptide. Furthermore, the system overcame the burst release commonly observed in conventional drug delivery, reducing side effects.

There is also great interest in improving the bioavailability of curcumin, as this polyphenol has powerful therapeutic properties in respiratory and liver diseases, diabetes and cancer. In this context, Altunbas and colleagues [57] combined this compound with MAX8 hydrogel in order to overcome solubility and degradation issues and to provide advantages such as minimally invasive, controlled and local administration. Since curcumin is neutral at physiological pH, the authors ascribed the changes in its post-encapsulation microenvironment to interactions with valine-rich faces in the core of the MAX8 gel fibres (to minimize water interactions). Although β-sheet content, β-hairpin folding, and post shear-thinning self-healing capacity of the fibre network were not affected by curcumin, the storage modulus G′ increased. This phenomenon can be explained by two mechanisms: (i) curcumin resides in the hydrophobic core of the fibres, reducing relaxation of hydrogen bonded fibrils and stiffening them; (ii) curcumin domains can bind different fibres, increasing the number of cross-links in the network. In this study, human medulloblastoma DAOY cells were used to test the therapeutic effect of MAX8 matrix-encapsulated curcumin. After 24 h of cell culture in 4 mM curcumin-loaded MAX8 gel, cell death occurred in and around the system, indicating the release of the therapeutic agent from the hydrogel network. Furthermore, cell death decreased with increasing peptide concentrations, suggesting that the stronger hydrophobic interactions between curcumin and MAX8 peptide slowed down drug release. This shows that not only the release profile can be tuned, but also that the released curcumin is biologically active. The authors state that the sustained drug release from MAX8 hydrogels eliminates the need for high concentrations of the compound, since the medium is continuously exposed to relevant doses of functional curcumin [57].

Years later, in a similar study, vincristine was incorporated into the three-dimensional network of MAX8 hydrogel to avoid its repeated intravenous administration and consequent side effects [58]. Interestingly, oscillatory rheology found that the presence of the drug in the hydrogel matrix does not induce any change in its mechanical behaviour. Thus, to obtain clues about the specific location of vincristine in the hydrogel, the authors performed SANS. The results showed that the morphology of the fibre network, whether it incorporates the drug, is similar; however, the slight increase in mid-q scattering intensity suggests that vincristine binds closely to and along the fibres rather than forming separate clusters or dispersing into water. Drug release studies revealed that encapsulated vincristine was released continuously for 28 days at low but effective concentrations, remaining active for a period approximately 20 times longer than in bulk water. Compared to intravenous administration, which exposes the entire body to the toxic effects of the compound, local delivery of vincristine with MAX8 hydrogel can provide local, low-dose, sustained treatment, reducing systemic toxicity.

## 5. From MAX8 to Its Analogues: Expanding the Design Space

Based on the success of MAX8 hydrogel, several analogous peptides have been developed with the aim of better understanding the properties of the respective materials and improving them, particularly in the biological and production sectors. By modifying or adding amino acids to the peptide sequence, the spectrum of applications broadens to include regenerative medicine and biotechnology, among others, and provides us with more knowledge of the structure-activity relationship that governs the performance of peptides and hydrogels. The following chapters are therefore concerned with the identification of MAX8 peptide analogues and the new insights they have brought to the field. All the peptide sequences are reported in Table 5.

### 5.1. Re-Designing MAX8 for Microbial Expression and Large-Scale Manufacturing

In an interesting work, three MAX8-derived peptides (**EX1**, **EX2**, and **EX3**) were de novo synthesized by replacing the original turn sequence with all L-amino acids and the terminal amide with a carboxylic acid [59] (all the sequences are reported in Table 5). These substitutions were carried to produce peptides with similar characteristics to MAX8 but capable of being synthesized by bacterial expression. EX1 peptide turn consisted of 5 residues -VPDGT- and was designed to adopt a 3:5 type 1 + G1 β-bulge turn (where hydrogen bonds are formed between the amide of valine and the carbonyl of threonine and between the carbonyl of valine and the amide of glycine). EX2 had the non-polar side chain residue isoleucine instead of aspartate at position *i* + 2 of the turn (-VPIGT-), thereby stabilizing the 3:5 type 1 + G1 β-bulge turn. EX3 had the turn sequence -YNFT- designed to adopt a 2:2 type I′ β-turn—like the type II′ of MAX8 and MAX1 but differing in main chain dihedral angles. CD data showed that all peptides self-assemble into a β-sheet-rich network, and TEM images revealed fibres with diameters of 3–4 nm, consistent with the previously determined length of a self-assembled β-hairpin [29,52,53]. These studies are extremely important as they prove that the folded conformation of a peptide is crucial in defining the local morphology of gel fibres. Oscillatory rheology experiments revealed that the EX1-formed hydrogel breaks at lower strains and recovers a lower percentage of elasticity after shear-thinning. The authors attributed these characteristics to the negatively charged-aspartic acid residue, which, being located on the hydrophobic face of the hairpin, may not be well accommodated within the fibre; this might produce imperfections that lead to the slight heterogeneity of the fibres and impact network rheometry. After optimizing the fusion patterns for stabilizing the expression of the designed peptides in *E. coli*, yields of 50, 31 and 15 mg/L of HPLC-purified of EX1, EX2 and EX3, respectively, were achieved [59]. The fact that these peptides have been shown to fold and self-assemble the same way as their synthetic counterparts demonstrates that it is possible to use a scalable approach to produce β-hairpin peptide hydrogels.

### 5.2. Programming Biological Function into MAX8 Hydrogels

Gungormus et al. [60] set out to use MAX8 hydrogel as a starting point to tackle the problem of repairing/replacing cartilage, bone, and dental tissue. In this context, the authors synthesized **MDG1**, a 27-residue peptide consisting of the MAX8 self-assembling 20-residue sequence followed by the 7-residue sequence -MLPHHGA-NH_2_—responsible for directing mineralisation in the presence of CaCl_2_, β-glycerophophate (β-GP) and phosphatase, when protruding from the fibres’ surface [60]. To study the effect of the seven-residue sequence on mineralisation, an analogue, cMDG1 was also synthesized: it was identical to MDG1 but had the 7-residue portion reversed. CD revealed that both peptides exhibit β-sheet secondary structure, albeit to a lesser extent than the MAX8 hydrogel. This indicates that the terminal peptide does not contribute to β-sheet folding, regardless of the sequence order. The analogues gelled in the presence of CaCl_2_ and β-GP, and mineralisation occurred in all cases. Nevertheless, SEM images revealed that the mineral deposited in the MDG1 hydrogel is considerably more crystalline than the one deposited in the cMGD1 hydrogel (which in turn is more crystalline than that deposited in the MAX8 hydrogel), indicating that the MLPHHGA sequence does not influence mineralisation, but rather the morphology of the mineral. Such observations were confirmed by XRD measurements [60]. Indeed, the ability to form hydrogels containing inherent functionalities makes these hybrid peptides promising candidates for developing smart materials for nanotechnological applications.

In 2017, MAX8 analogues containing extracellular matrix adhesion motifs, such as RGDS (derived from fibronectin, forming **MAX8-RGDS**), IKVAV (derived from laminin, forming **MAX8-IKVAV**) and YIGSR (derived from laminin, forming **MAX8-YIGSR**) were explored for high throughput screening (HTS) applications [61]. While MAX8 hydrogel maintained medulloblastoma cells viable, gels formed from functionalised peptides increased cell proliferation, allowed long-term cell maintenance and helped them take on a phenotype similar to that of cancer tissue. These findings show how the rational design and modification of existing peptides is biologically instructive, offering a customisable platform for the discovery of anticancer drugs [61].

### 5.3. Designing for Precision: Electrostatics and Gelation/Drug Release Kinetics in Self-Assembling Peptides

Shortly after the design of MAX8, interest arose in understanding how small changes in peptide sequence impact the kinetics of gelation and fibrillar network formation. To this end, two analogues were synthesized which, like MAX8, differ from MAX1 in the amino acid residue at position 15: **MTHR** (containing a threonine residue) and **MGLN** (containing a glutamine residue) [54]. The study was conducted using microrheology to measure the viscoelastic properties of the peptide solution as it gels. It was found that MAX8 peptide gels the fastest due to the favourable electrostatic attraction between the glutamate and lysine residues at room temperature (25 °C). MTHR and MGLN, which feature polar but uncharged side chain residues, have longer gelation times; however, rising the temperature to 35 °C increases the diffusivity of the monomers and decreases the system organization according to the hydrophobic character of its components, causing the peptides to assemble at a faster rate. Ultimately, MAX1 presents the greatest energy barrier to overcome, requiring the highest temperature to achieve a gelation time comparable to that of other peptides: 50 °C. The authors further learnt that the gelation time determined by microrheology matches the onset formation of β-sheets. Indeed, CD measurements revealed that, for all peptides, gelation occurs when the minimum mean-residue ellipticity at a wavelength of 216 nm reaches a value of −24 × 10^3^ and −22 × 10^3^ deg dmol^−1^ cm^2^ [54]. Thus, this work not only proves that a single modification in a hydrogelator peptide sequence changes its folding energy (and, consequently, the gelation time and response to stimuli) but also provides a quantitative link between secondary structure formation and macroscopic gel properties.

In a similar approach, Yamada and co-workers [62] explored how electrostatic complementarity between the payload, in this case the anticancer protein TIMP-2, and the hydrogel’s peptide network influences the former’s release kinetics and bioactivity preservation [62]. To this end, in addition to the classic MAX8 (charge of +7), they designed three gel-forming peptide analogues with different formal charges: **HLT2** (formal charge of +5), **AcVES3** (formal charge of −5) and **IE1** (formal charge of −7). Rheological properties, particularly the self-healing capacity, of each hydrogel encapsulating 4 mg/mL of TIMP-2 were evaluated, and it was found that the protein hinders the post-shear-thinning recovery of the hydrogels formed by positively charged peptides (MAX8 and HLT2). In addition, protein release exceeded 80% in the latter only after three days of monitoring; in contrast, it reached 40% in AcVES3 and IE1-formed gels, remaining linear and sustained over the following 25 days, as intended. Although the pI of TIMP-2 is 6.84 (approximately neutral at physiological pH), the protein has positively charged-residues on the surface, making electrostatic interactions between them and the fibres quite probable. Thus, repulsion is likely to occur between TIMP-2 and the MAX8 and HLT2 fibres, while favourable interactions are expected between TIMP-2 and the AcVES3 and IE1 fibres. Given the optimal rheological properties of the AcVES3/TIMP-2 complex, studies regarding encapsulated TIMP-2 activity were carried using that system. CD results confirmed the retention of protein folding, and kinetic analysis showed the maintenance of its activity in suppressing the proliferation of A549 lung cancer cells [62]. This study highlights the importance of electrostatic interactions in modulating drug delivery kinetics and suggests exploring this concept to fine-tune delivery systems for other proteins/drugs.

By changing the turn peptide sequence, electrostatic interactions, or coupling bioactive motifs, the scientific community turned its attention to gelation and cargo release kinetics as well as specific gel-tissue interactions. However, the diversity of new β-hairpin-forming molecules has introduced a high degree of complexity that makes comparison difficult, as even the smallest change affects several parameters simultaneously. This makes isolating cause-effect (structure-activity relationship) the greatest challenge in this field. Ultimately, this chapter highlights the potential, but also the fragility, of the β-hairpin design, suggesting the need for systematic approaches that drive the development of analogues beyond the empirical (trial-and-error) approaches we have seen.

## 6. Other β-Hairpin Peptides (Non-MAX Peptides)

**Figure 12:**
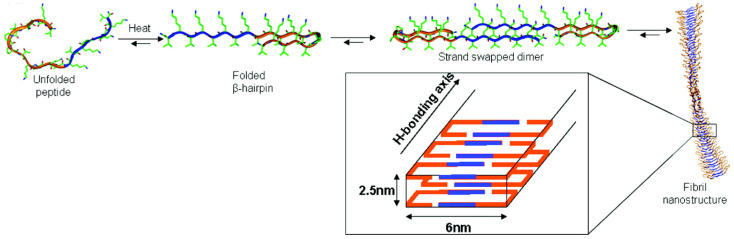
Proposed self-assembly mechanism of the strand-swapping peptide SSP1. Adapted with permission from Ref. [64].

Besides MAX1 and MAX8, other *β*-hairpin peptides have also been described for the development of hydrogels. The pioneering “*non-MAX*” peptides included derivatives of these same structures (all the sequences are reported in Table 6). For instance, Nagarkar et al. [64] found that the 20-residue strand-swapping peptide 1 (SSP1) could adopt a soluble random coil conformation at pH 9 and temperatures less than 35 °C, whereas heating to 37 °C or higher would lead to the formation of a β-hairpin with an exchangeable β-strand region. The exchangeable β-strand domain, comprising 8 residues, could participate in swapping with the exchangeable domain of another peptide, leading to strand-swapped dimers, further assembling in fibres and the formation of a hydrogel (Figure 12). A smaller exchangeable β-strand domain (SSP2) also led to the same behaviour, and it could directly influence the fibril nanostructure, morphology and rheology. In the case of SSP1 gels, the network consisted of twisted fibres with a diameter of 6.4 nm, height of 6.0 nm, and pitch of 50.4 nm, while SSP2 displayed non-twisted fibres of 6.2 nm in diameter and 2.5 nm in height. Consequently, the mechanical properties of the gels were also different, with the SSP2 gels forming at a faster rate than SSP1 and affording a larger G′ (517 Pa compared to 367 Pa in SSP1). Later, the same group [65], assessed the role of strand asymmetry using the peptide SSP3, in which the strand registry is switched relative to SSP2 such that the *N*-terminal strand is exchanged instead of the *C*-terminal strand. The SSP3 gels comprised a network of laminated fibrils, achieving a larger storage modulus (2739 ± 191 Pa) than SSP1 and SSP2 gels, which the authors attributed to the stiffer fibrillar morphology of SSP3 compared to the flexible fibrils obtained in the other peptide gels.

By employing the type II′ β-turn region “V^D^PPT” and stimuli-responsive moieties, metal and light-triggered β-hairpin peptide-based gels can be developed. Rughani et al. [67] developed a photopolymerizable 22-residue peptide, **MLD**, which could undergo temperature-induced folding and self-assembly, forming moderately rigid, shear-thinning, and reversible hydrogels with a G′ ~220 ± 50 Pa (1.0 wt%). Upon light irradiation of the gel network, the non-natural sorbamide derivatives of lysine could lead to the polymerization of dienes on the surface of the fibrils, resulting in an increase in the gel’s mechanical rigidity ~2.5-fold. Later, Schneider’s group reported a β-hairpin peptide (**ZnBHP**) [66], in which zinc binding leads to the peptide folding, followed by self-assembly into β-sheet-rich fibrils and formation of a gel. The metal responsiveness was conferred by placing a negatively charged unnatural metal-binding amino acid, 3-amidoethoxyaminodiacetoxy-2-aminopropionic acid, at the *C*-terminus at position 20. Upon chelation of Zn^2+^ ions, the side chain becomes neutral, reducing the energy barrier for folding, thus enabling β-hairpin formation, with the metal-binding amino acid becoming desolvated and incorporated within the hydrophobic environment of the folded state. The gel displayed a G′ of 157 ± 43 Pa after 2 h at 30 °C, with a network characterized by physically cross-linked entangled fibrils with a width of 3 nm, in which the formation of fibrillar lamination could also be observed, with the fibrils being stacked or twisted around each other.

Peptides based on the type II′ β-turn region “V^D^PPT” have also been explored for combination with enzymes, or for enzyme responsiveness. Li et al. [72] reported gels based on 20-residue hairpin peptides loaded with glucose oxidase, and catalase for stimuli-responsive insulin release. The strategy involves, under hypoglycaemia, the enzymatic conversion of glucose to gluconic acid leading to a decrease in local pH, which results in the repulsion of neighbouring alkaline amino acid side chains, and consequently, on the unfolding of the hairpins, disassembly, and insulin release. Among the gels, the **IA-0** and **IA-2** displayed the best responsiveness, in which the replacement of valine with isoleucine was found to result in a faster gelation kinetics and larger storage modulus (IA-2: G′ ~1000 Pa vs. IA-0: G′ ~400 Pa). Giano et al. [68] developed 20-amino acid degradable peptides (**DP**) by incorporating an MMP-13-cleavable, six-residue sequence (PTG-XKV, X = A, F, I, L) at the *C*-terminus. The gels could be formed under physiologically relevant conditions (150 mM NaCl, pH 7.6), and specifically degraded by MMP-13, with the susceptibility depending on the cleavage site amino acid sequence (X = F > L > I > A). Additionally, in vitro assays revealed that the SW1353 cells could invade into and migrate through the gels’ matrices, highlighting the potential of this strategy for facilitating repair of damaged tissues that have elevated levels of MMP-13.

Aiming to achieve biological activity, Veiga et al. [70] developed β-hairpin peptides with a high content of arginine (**PEP6R**), which could form gels with G′ ~1200 Pa upon addition of a buffer containing NaCl. Notably, the authors found that 6 residues of arginine could result in the strongest gels among the developed structures. In addition, the gels were strongly effective at killing both Gram-positive and negative bacteria, including the multi-drug-resistant Pseudomonas aeruginosa, while presenting cytocompatibility towards human erythrocytes and mammalian mesenchymal stem cells.

Despite most of the advancements with the type II′ β-turn region “V^D^PPT”, other moieties and peptide sequences have also been explored, yet to a lesser extent. For instance, Chen et al. [73] designed several 16-residue amphiphiles in which the cation-π interaction pairs achieved by the incorporation of tryptophan drive the folding into a β-hairpin conformation, and subsequent self-assembly into fibril-rich hydrogels. Among the synthesized peptides, YT-W (X_1_ = W, X_2,3_ = K) could quickly form gels, reaching 115 Pa at 60 min after inducing gelation with BTP buffer. Doran et al. [71] also reported a photo-responsive β-hairpin peptide consisting of **(RADA)_4_** incorporated with [3-(3-aminomethylphenylazo)phenyl]acetic acid (AMPP) as a light-responsive modulator of the peptide secondary structure. In the trans state, AMPP is in a β-arc conformation, forming gels with a G′ value of 260 ± 60 Pa, while in the cis conformation, the peptide adopted a type I′ β-hairpin, leading to a weakening of the hydrogel to a G′ value of 80 ± 20 Pa. Additionally, as the authors founds that the gel could recover upon reverse cis–trans photoisomerization, the loss of rigidity was associated with the disruption of the well-ordered macromolecular structure instead of the disassembly of the fibrils. Regarding metal-responsive gels, Knerr et al. [69] developed a 20-residue peptide (**MBHP**) that could form hydrogels upon heavy metal ions complexation in a 1:1 metal-peptide stoichiometry (monomethylarsonous acid (MMA), Pb^2+^, Zn^2+^, Cd^2+^ or Hg^2+^) through two cysteine residues on opposing ends of the β-turn. The authors found that the gelation kinetics depended on the metal ion, with the MMA-, Zn^2+^-, and Hg^2+^-triggered gelation starting within the first minute, while Cd^2+^ and Pb^2+^-triggered gels took 5 and 20 min to start gelation, respectively. Additionally, the storage modulus of the gels was influenced by the metal ion as follows: Zn^2+^ (4140 ± 330 Pa) > Cd^2+^ (1960 ± 750 Pa) > MMA (1720 ± 120 Pa) > Pb^2+^ (1210 ± 330 Pa) > Hg^2+^ (G′ 1000 ± 510 Pa). Further analysis of MMA- and Zn^2+^-gels revealed that the networks were composed of elongated, entangled fibrils (~3 nm) with a regular twist and in some cases, high order laminates, which is also commonly observed for β-hairpin peptides containing the β-turn region “V^D^PPT”.

Other peptide structures with biological activity were also reported. Liu et al. [74] reported the **ASCP1**, comprising the antibacterial peptide sequence (KIGAKI)_3_-NH_2_ and a central tetrapeptide linker (-T^D^PPG-), which exhibits promising antibacterial activity against *Escherichia coli*. The peptide could self-assemble into a gel when exposed to different external stimuli, such as pH, ionic strength and temperature. Notably, the authors found that the gel displayed thermally reversible behaviour, in which increasing the temperature from 20 °C to 65 °C would lead to an increase in storage modulus from ~20 Pa to ~4000 Pa. Furthermore, the inherent antibacterial activity of the peptide hydrogel was confirmed by the antibacterial assay against *Escherichia coli.* Considering the potential of α-helical oncolytic peptide-mediated immunotherapy, which is limited by the proteolytic vulnerability of linear peptides, Lu et al. [75] developed a β-hairpin oncolytic peptide (**TPI-Se**) stabilized by double diselenide bonds, derived from the natural peptide Tachyplesin I. The peptide was incorporated into injectable and self-healing sodium alginate-based gels with G′ > 10,000 Pa. The authors found that the incorporation of diselenide bonds enhanced the peptide’s proteolytic and reductive stability, compared to cysteine-containing peptides, as well as the oncolytic immunotherapeutic efficacy. Additionally, the co-loading of gels with an immune checkpoint inhibitor (PD-L1 inhibitor, ^D^PPA-1) further enhanced the antitumor immunity by remodelling the tumour immunosuppressive microenvironment.

Recently, Pizzella et al. [76] proposed a procedure to identify from structural data available in the Protein Data Bank (PDB) novel aggregation-prone peptide fragments. This new approach was applied to fragments of the self-assembling protein transthyretin (TTR). Modification and mutation of TTR sequences (e.g., mutation Val50Met) are related to the generation of amyloid-like variants, thus producing inherited transthyretin amyloidosis. Specifically, **TTR^34–52^** was found as a stable β-harpin forming sequences, formed by two interacting peptides, composed by residues 34–40 (**TTR^34–40^**) and 45–52 (**TTR^45–52^**). Molecular Dynamics simulation suggested a good stability suggesting the use of TTR^34–52^, TTR^34–40^, and TTR^45–52^ as novel self-assembling sequences. All the peptides were found able to form self-supporting hydrogelated matrices, including the co-assembly system TTR^34–40^+TTR^45–52^. The proposed sequences also demonstrated photoluminescent behaviour at solid state.

## 7. Conclusions

MAX1 and its analogues represent a paradigmatic class of de novo-designed self-assembling peptides for β-hairpin-based hydrogels generation. These matrices are characterized by highly tuneable and predictable properties as consequence of precise control over peptide secondary structure, fibrillar morphology, gelation kinetics, and mechanical response. MAX1 was consequently a model peptide for elucidating structure–property–function relationships in supramolecular biomaterials. Compared with ultra-short or modified natural peptides, MAX1 offers greater modularity and responsiveness to external stimuli, including pH, light, oxidation, and ionic strength.

Collectively, the reported studies identify hydrophobicity and electrostatic interactions as the key molecular parameters governing self-assembly and material performance. Even minimal changes in hydrophobicity significantly influence gelation kinetics, thermal reversibility, mechanical rigidity, and fibril architecture; however, hydrophobicity alone is insufficient to predict gel formation. In turn, amino acid modification alters the electrostatic peptide features, demonstrating that charge balance critically affects peptide folding and hydrogel stability. These findings highlight that a finely tuned balance of both hydrophobic and electrostatic interactions is mandatory to obtain stable and functional β-hairpin-based hydrogels.

An important outcome derived from MAX1-derived designs is the demonstration that molecular engineering can translate external stimuli (including pH, light, oxidation state, or mechanical stress) into dynamic and reversible material responses. In parallel, strategies aimed at controlling fibril morphology and supramolecular architecture revealed that increased structural order does not necessarily result in improved macroscopic and rheological properties, underscoring the need to align architectural design with application-driven performance requirements.

MAX8 further validates the β-hairpin design principle and reinforces the relationship between peptide sequence, nanoscale structuration, and matrix performances. While scattering and rheological studies have clarified aspects of fibre packing and cross-linking, the partial loss of elasticity observed during repeated shear-thinning cycles raises concerns regarding the long-term mechanical stability of these materials under physiologically relevant dynamic conditions.

Type II′β-turn motif (VDPPT) or alternative β-hairpin promoting sequences were explored as structural elements for gelation, thereby expanding the design space beyond MAX1 and MAX8. Structure–property studies suggested that strand exchange length and asymmetry (*N*- versus *C*-terminal exchange) strongly influence both fibril supramolecular organization and rheological response. Sequence modifications enable further control over gelation kinetics and mechanical properties. Additional strategies include enzyme encapsulation, incorporation of cleavable sequences for stimuli-responsive behaviour, and functionalization with biologically active or bioactive motifs (e.g., antibacterial arginine-rich or oncolytic sequences).

Overall, β-hairpin peptide hydrogels provide a robust and versatile framework for tuning macroscopic material properties through molecular design, positioning these systems as valuable prototypes for the development of responsive, biocompatible platforms for biomedical and technological applications.

Despite the promising in vitro results regarding the cytocompatibility and mechanical responsiveness of MAX1 and its analogues matrices, it is crucial to recognize the inherent limitations about prediction of long-term behaviour in complex biological systems. While in vitro evidence demonstrates that these hydrogels can maintain their structural integrity and support cell proliferation, no sufficient data are collected for more complex in vivo environments, including multimodal interplay of proteolytic enzymes, fluctuation of local pH gradients, and dynamic immune cell interactions with the matrices. Furthermore, although short-term assays show minimal pro-inflammatory responses, the long-term chronic immune response and the eventual metabolic fate of the degraded peptide fragments remain areas of significant uncertainty. Additionally, the shear-thinning and self-healing nature of these materials, while ideal for injection, introduces questions regarding their long-term mechanical stability in high-strain physiological environments where continuous movement may lead to domain fracture and reduced network rigidity over time. As evident also for other peptide-based systems, bridging the gap between these controlled in vitro observations and clinical translation will require more sophisticated, long-term in vivo approach and studies to fully understand the life cycle of these beta-hairpin scaffolds within a living organism.

As for other peptide-based hydrogels [77], the evolution of β-hairpin ones, driven by the MAX1 and MAX8 paradigms, has reached a critical point. The focus should move from “trial-and-error” synthesis to a non-empirical and predictive design approach. Strengthening a priori knowledge about mechanistic understanding requires a deeper learning of folding-coupled assembly. To move toward practical and non-empirical design, future research should focus on additional knowledge, including deterministic sequence control, via quantification of lateral hydrophobic interactions contributions, the chirality rule as a molecular tool for aggregation, industrial scalability and long-term stability, and in vivo integration.

## Figures and Tables

**Table 1:** Hydrogels based on MAX1 and MAX1-analogue peptides, showing substitutions of one or more residues (highlighted in bold) to modulate gelation temperature. Below, self-assembly parameters, gelation temperature, elastic modulus (G′) and fibril cross-section are reported. n.r. = not reported; [d] = diameter; [w] = width.

Gelator	Amino Acid Sequence	Gelation Conditions	T_gel_ (°C)	G′ (Pa)	Fibril (nm)	Ref#
**MAX1**	VKVKVKVKV^D^PPTKVKVKVKV-NH_2_	2 wt%, 125 mM Borate, 10 mM NaCl, pH 9	~25	~1100	~3 [d]	[18]
**MAX2**	VKVKVKVKV^D^PPTKVK**T**KVKV-NH_2_	2 wt%, 125 mM Borate, 10 mM NaCl, pH 9	~40	n.r.	n.r.	[36]
**MAX3**	VKVKVK**T**KV^D^PPTKVK**T**KVKV-NH_2_	2 wt%, 125 mM Borate, 10 mM NaCl, pH 9	~60	~1100	n.r.	[36]
**MAX4**	**KVKVKVKVK**^D^PP**SVKVKVKVK**-NH_2_	1 wt%, 125 mM Borate, 10 mM NaCl, pH 9	~26	~250	n.r.	[37]
**MAX5**	VKVKVKVKV^D^PP**S**KVKVKVKV-NH_2_	1 wt%, 125 mM Borate, 10 mM NaCl, pH 9	~25	~1000	~3 [d]	[37]
**MAX1I4**	**I**K**I**KVKVKV^D^PPTKVKVK**I**K**I**-NH_2_	2 wt% [25 mM Hepes, pH 7.4/DMEM (1/1)]	20 ± 2	492	~3 [w]	[38]
**MAX1I4′**	VKVK**I**K**I**KV^D^PPTK**I**K**I**KVKV-NH_2_	2 wt% [25 mM Hepes, pH 7.4/DMEM (1/1)]	20 ± 2	942	~3 [w]	[38]
**MAX1I8**	**I**K**I**K**I**K**I**KV^D^PPTK**I**K**I**K**I**K**I**-NH_2_	2 wt% [25 mM Hepes, pH 7.4/DMEM (1/1)]	20 ± 2	1494	~3 [w]	[38]
**M(Abu)**	**X**K**X**K**X**K**X**KV^D^PPTK**X**K**X**K**X**K**X**-NH_2_ **X** = (Aminobutyric acid)	1 wt%, 125 mM Borate, 10 mM NaCl, pH 9	70	~1090	n.r.	[39]
**M(Nva)**	**X**K**X**K**X**K**X**KV^D^PPTK**X**K**X**K**X**K**X**-NH_2_ **X** = (Norvaline)	1 wt%, 125 mM Borate, 10 mM NaCl, pH 9	30	3300	3.3 [w]	[39]
**M(Nle)**	**X**K**X**K**X**K**X**KV^D^PPTK**X**K**X**K**X**K**X**-NH_2_ **X** = (Norleucine)	1 wt%, 125 mM Borate, 10 mM NaCl, pH 9	~5	680	3.2 [w]	[39]
**M(Phe)**	**F**K**F**K**F**K**F**KV^D^PPTK**F**K**F**K**F**K**F**-NH_2_	1 wt%, 125 mM Borate, 10 mM NaCl, pH 9	n.r.	n.r.	3.4 [w]	[39]
**M(Ile)**	**I**K**I**K**I**K**I**KV^D^PPTK**I**K**I**K**I**K**I**-NH_2_	1 wt%, 125 mM Borate, 10 mM NaCl, pH 9	n.r.	n.r.	3.3 [w]	[39]

**Table 2:** Hydrogels based on MAX1 and MAX1-analogue peptides, showing substitutions of one or more residues (highlighted in bold) to modulate net charge. Below, net charge at neutral pH (pH 7), elastic modulus (G′) and fibril cross-section are reported. n.r. = not reported; [d] = diameter; [w] = width.

Gelator	Amino Acid Sequence	Charge	Gelation Conditions	G′ (Pa)	Fibril (nm)	Ref#
**MAX1(K15R)**	VKVKVKVKV^D^PPTKV**R**VKVKV-NH_2_	+9	0.5 wt% in 50 mM buffer pH 8 + 10 mM NaCl	~50	~3 [w]	[40]
**MAX1(K15T)**	VKVKVKVKV^D^PPTKV**T**VKVKV-NH_2_	+8	0.5 wt% in 50 mM buffer pH 8 + 10 mM NaCl	~200–300		[40]
**MAX1(K15I)**	VKVKVKVKV^D^PPTKV**I**VKVKV-NH_2_	+8	0.5 wt% in 50 mM buffer pH 8 + 10 mM NaCl	~200–300		[40]
**MAX1(K15E)**	VKVKVKVKV^D^PPTKV**E**VKVKV-NH_2_	+7	0.5 wt% in 50 mM buffer pH 7.4 + 10 mM NaCl	~600		[40]
**MAX1(K4E, K15E)**	VKV**E**VKVKV^D^PPTKV**E**VKVKV-NH_2_	+5	0.5 wt% in 50 mM buffer pH 8 + 10 mM NaCl	unstable		[40]
**MAX1(V16E)**	VKVKVKVKV^D^PPTKVK**E**KVKV-NH_2_	+8	None	None	None	[40]
**MARG1**	VKVKV**R**VKV^D^PPTKVKV**R**VKV-NH_2_	+8	2 wt% in H_2_O + DMEM (1:1 *v*/*v*)	~2200 Pa	n.r.	[41]
**Peptide 1**	VKVKV**H**V**H**V^D^PPT**H**V**H**VKVKV-NH_2_	n.r.	1 wt% in 50 mM Tris+ 10 mM ZnCl_2_; 1 wt% in 50 mM Tris+ 30 mM NaCl	~170 Pa (ZnCl_2_) ~180 Pa (NaCl)	~3.2–3.8 [d]	[42]
**VX1**	VXVXVXVXV^D^PPTXV**X**VXVXV-NH_2_ **X** = aminoadipic acid	−7	0.5 wt% in 50 mM BTP + 150 mM NaCl	n.r.	~3 [w]	[43]
**VE1**	V**E**V**E**V**E**V**E**V^D^PPT**E**V**E**V**E**V**E**V-NH_2_	−7	0.5 wt% in 50 mM acetate/BTP + 150 mM NaCl	n.r.	~3 [w]	[43]
**VD1**	V**D**V**D**V**D**V**D**V^D^PPT**D**V**D**V**D**V**D**V-NH_2_	−7	0.5 wt% in acetate+ 150 mM NaCl	~1500	~3 [w]	[43]
**VE3**	V**E**VKV**E**V**E**V^D^PPT**E**VKV**E**V**E**V-NH_2_	−3	0.5 wt% in 12.5% HEPES + 75 mM NaCl	1386 ± 162	n.r.	[43]
**VEQ1**	V**Q**V**E**V**Q**V**E**V^D^PPT**Q**V**E**V**Q**V**E**V-NH_2_	−3	0.5 wt% in 12.5% HEPES + 75 mM NaCl	1463 ± 91	n.r.	[43]
**CBHH**	VKVKV**H**V**H**V^D^PPT**H**V**H**VKVKV-NH_2_	n.r.	4 mM in Phosphate buffer 15 mM pH 5.7+ SA 4 mM; 4 mM in Phosphate buffer 15 mM pH 5.7+ MA 4 mM; 4 mM in Phosphate buffer 15 mM pH 5.7+ TA 4 mM	CBHH + SA ~350 CBHH + MA ~470 CBHH + TA ~100	2–4 [d]	[44]
**H4LMAX**	**LHLHL**K**L**KV^D^PPTK**L**K**LHLHL**-NH_2_	+5	1 wt% in 50 mM Tris + 30 mM NaCl	210	~3.6 [w]	[45]
**H4LMAX-RGDS**	**LHLHL**K**L**KV^D^PPTK**L**K**LHLHL**-**RGDS**	+5	1 wt% in 50 mM Tris + 30 mM NaCl	40	~4.9 [w]	[45]
**H2LRDMAX**	**LHLNL**K**L**KV^D^PPTK**L**K**LRLHL**-NH_2_	+5	1 wt% in 50 mM Tris + 30 mM NaCl	510	~3.6 [w]	[45]

**Table 3:** Hydrogels based on MAX1 and MAX1-analogue peptides, showing substitutions of one or more residues (highlighted in bold). The modification induces a stimuli-induced gelation. Triggers are reported. Below, self-assembly parameters, gelation trigger, elastic modulus (G′) and fibril cross-section are reported. n.r. = not reported; [d] = diameter.

Gelator	Amino Acid Sequence	Gelation Conditions	Gelation Trigger	G′ (Pa)	Fibril (nm)	Ref#
**MAX6**	VKVKVKVKV^D^PPTKVK**E**KVKV-NH_2_	No hydrogel	No hydrogel	-	n.r.	[46]
**MAX7**	VKVKVKVKV^D^PPTKVK**C**KVKV-NH_2_	2 wt%, 125 mM Borate, 10 mM NaCl, pH 9	pH	~1100	n.r.	[46]
**MAX7CNB**	VKVKVKVKV^D^PPTKVK**X**KVKV-NH_2_ **X** = Cys with 2-nitrobenzyl photocage	2 wt%, 125 mM Borate, 10 mM NaCl, pH 9 + UV light (λ > 300 nm)	light	~1000	n.r.	[46]
**VK20**	**CH_2_=C(CH_3_)−CO−NH−** VKVKVKVKV^D^PPTKVKVKVKV−NH_2_	Cryopolymerized into PEG network at −20 °C; pH-responsive	pH	~1000	n.r.	[47]
**Tyrosine-functionalized analogue**	VKVKVKVKV^D^PPTKV**Y**VKVKV-NH_2_	1 wt% in HEPES buffer, oxidized post-assembly	Oxidation by Frémy’s salt	~25,470	~3 [d]	[48]

**Table 4:** Hydrogels based on MAX1 and MAX1-analogue peptides, showing substitutions of one or more residues (highlighted in bold) to modulate backbone flexibility and fibril bundling/branching behaviour. Below, self-assembly parameters, gelation trigger, elastic modulus (G′) and fibril cross-section are reported. n.r. = not reported; [d] = diameter; [w] = width.

Gelator	Amino Acid Sequence	Gelation Conditions	G′ (Pa)	Fibril (nm)	Ref#
**LNK1**	(**Nal**)K(**Nal**)K**A**K**A**K-V^D^PPT-K**A**K**A**K(**Nal**)K(**Nal**)-NH_2_	2 wt%, pH 9, 125 mM boric acid, 10 mM NaCl	~200 → ~5	~3 [w]	[49]
**P1/GP1**	VKVKVKVKV**FANGT**VKVKVKVKV-NH_2_ VKVKVKVKVFAN*GTVKVKVKVKV-NH_2_	1 wt%, pH 7.4, 37 °C	P1: 824 ± 51 GP1: 284 ± 59	P1: ~4 [w] GP1: ~4.5 [w]	[50]
**P2/GP2**	VKVKVKVK**FANGT**KVKVKVKV-NH_2_ VKVKVKVKFAN*GTKVKVKVKV-NH_2_	1 wt%, pH 7.4, 37 °C	P2: 109 ± 18 GP2: 18 ± 7	P2: ~4.5 [w] GP2: ~4.8 [w]	[50]
**P3/GP3**	VKVKVKVKV**YNGT**VKVKVKVKV-NH_2_ VKVKVKVKVYN*GTVKVKVKVKV-NH_2_	1 wt%, pH 7.4, 37 °C	P3: 637 GP3: 364	P3: ~4.5 [w] GP3: ~4.9 [w]	[50]
**P4/GP4**	VKVKVKVK**YNGT**KVKVKVKV-NH_2_ VKVKVKVKYN*GTKVKVKVKV-NH_2_	1 wt%, pH 7.4, 37 °C	P4: 309 ± 25 GP4: 243 ± 24	P4: ~4.1 [w] GP4: ~4.4 [w]	[50]
**P5/GP5**	VKVKVKVKV**FANGT**VKV**E**VKVKV-NH_2_ VKVKVKVKVFAN*GTVKV**E**VKVKV-NH_2_	pH 7.4, 37 °C	P5: 518 ± 186 GP5: 927 ± 177	P4: ~4.2 [w] GP4: ~4.7 [w]	[50]

**Table 5:** Hydrogels based on MAX8 and MAX8-analogue peptides. Modifications in amino acid sequence are highlighted in bold. Below, self-assembly parameters, gelation pH, elastic modulus (G′) and fibril cross-section are reported. n.r. = not reported; [d] = diameter.

Gelator	Amino Acid Sequence	Gelation Conditions	pH	G′ (Pa)	Fibril (nm)	Ref#
**MAX8**	VKVKVKVKV^D^PPTKVEVKVKV-NH_2_	50 mM BTP buffer, 150 mM NaCl	7.4	270	~3.0 [d]	[52]
**EX1**	VKVKVKVKV**PDG**TKVEVKVKV-CO_2_H	50 mM BTP buffer, 150 mM NaCl	7.4	~1000	3–4 [d]	[59]
**EX2**	VKVKVKVKV**PIG**TKVEVKVKV-CO_2_H		7.4	~1000	3–4 [d]	
**EX3**	VKVKVKVK**YNG**TKVEVKVKV-CO_2_H		7.4	~1000	3–4 [d]	
**MDG1**	VKVKVKVKV^D^PPTKVEVKVKV-**MLPHHGA-**NH_2_	25 mM Tris buffer, 48 mM CaCl_2_, 28.8 mM β-GP	7.4	510	n.r.	[60]
**cMDG1**	VKVKVKVKVDPPTKVEVKVKV-**AGHHPLM**-NH_2_		7.4	590	n.r.	
**MAX8-RGDS**	**RGDS**VKVKVKVKV^D^PPTKVEVKVKV-NH_2_	50 mM HEPES buffer, DMEM	7.4	Unfinished kinetics	n.r.	[61]
**MAX8-IKVAV**	**IKVAV**VKVKVKVKV^D^PPTKVEVKVKV-NH_2_		7.4	n.r.	n.r.	
**MAX8-YIGSR**	**YIGSR**VKVKVKVKV^D^PPTKVEVKVKV-NH_2_		7.4	n.r.	n.r.	
**MTHR**	VKVKVKVKV^D^PPTKV**T**VKVKV-NH_2_	50 mM BTP	8.5	n.r.	n.r.	[54]
**MGLN**	VKVKVKVKV^D^PPTKV**Q**VKVKV-NH_2_		8.5	n.r.	n.r.	
**HLT2**	V**LT**KVK**T**KV^D^PPTKVEVKV**L**V−NH_2_	25 mM HEPES, 150 mM NaCl, 4 mg/mL TIMP-2	7.4	>1000	3 [d]	[62,63]
**AcVES3**	**Ac**−V**E**V**S**V**S**V**E**V^D^PPT**E**V**S**V**E**V**E**V−NH_2_		7.4	>1000	n.r.	
**IE1**	**IEIEIEIE**V^D^PPT**EIEIEIEI**-NH_2_		7.4	>1000	n.r.	

**Table 6:** Hydrogels based on other β-hairpin-forming peptides. Modifications in amino acid sequence are highlighted in bold Below, self-assembly parameters, gelation pH, elastic modulus (G′) and fibril cross-section are reported. n.r. = not reported; [d] = diameter; [w] = width; [h] = height; [p] = pitch.

Gelator	Amino Acid Sequence	Gelation Conditions	pH	G′ (Pa)	Fibril (nm)	Ref#
**SSP1**	VKVKV^D^PPTKV**K**VKVKV**KVKV**-NH_2_	125 mM borate, 10 mM NaCl	9	367	6.4 [d] 6.0 [h] 50.4 [p]	[64]
**SSP2**	VKVKVKV^D^PPTKV**K**VKVKV**KV**-NH_2_			517	6.2 [d] 2.5 [h]	
**SSP3**	**VK**VKVKVKVKV^D^PPTKV**K**VKV-NH_2_			2739	n.r.	[65]
**ZnBHP**	**I**KVK**I**KVKV^D^PPTK**I**KVK**I**K-**1**-NH_2_	1.5 mM ZnCl_2_, 50 mM BTP, 50 mM NaCl	7.4	157	~3 [w]	[66]
**MLD**	**K**VKV**-lysilsorbamide-**VKVKV^D^PPTKVKVXVKVK-**CO_2_H**	50 mM BTP, 150 mM NaCl, 0.025 wt% Irgacure	7.4	220	n.r.	[67]
**DP1**	**I**KVK**I**KVKV^D^PPT**GF**KVK**I**KV-NH_2_	50 mM Tris, 150 mM NaCl, 20 mM CaCl_2_, 40 mM ZnSO_4_	7.6	~1000	n.r.	[68]
**DP2**	**I**KVK**I**KVKV^D^PPT**GL**KVK**I**KV-NH_2_			~2500	n.r.	
**DP3**	**I**KVK**I**KVKV^D^PPT**GI**KVKIKV-NH_2_			~4000	n.r.	
**DP4**	**I**KVK**I**KVKV^D^PPT**GA**KVK**I**KV-NH_2_			~2000	n.r.	
**^D^** **DP3**	^D^(**I**KVK**I**KVKV)^**L**^P^**D**^(PT**GI**KVK**I**KV)-NH_2_			~4000	n.r.	
**MBHP**	VKVKVKV-**C**-**GPKE**-**C**-VKVKVKV-NH_2_	50 mM Tris, 150 mM NaCl, 1 eq. Metal salt	7.4	~1720	3 [w]	[69]
				~4140	2.9 [w]	
				~1960	n.r.	
				~1000	n.r.	
				~1210	n.r.	
**PEP2R**	VKVKVKV**R**V^D^PPT**R**VKVKVKV-NH_2_	50 mM BTP, 150 mM NaCl	7.4	~600	n.r.	[70]
**PEP4R**	VKVKV**R**V**R**V^D^PPT**R**V**R**VKVKV-NH_2_			~900	n.r.	
**PEP6R**	VKV**R**V**R**V**R**V^D^PPT**R**V**R**V**R**VKV-NH_2_			~1600	n.r.	
**PEP8R**	V**R**V**R**V**R**V**R**V^D^PPT**R**V**R**V**R**V**R**V-NH_2_			~1200	n.r.	
**Peptide 1**	(RADA)_4_-AMPP-(RADA)_4_ (**see referenced article**)	20 mM NaCl	-	260	3.4 [d]	[71]
**IA-2**	**I**K**I**K**I**K**I**K**I**^D^PPTK**IOI**K**I**K**I**-NH_2_	150 mM NaCl, Catalase, Glucose oxidase, Insulin	7.4	~1000	n.r.	[72]
**YT-W**	VKV**W**VK**Y**^D^**NG**TKV**K**VKV-NH_2_	50 mM BTP, 150 mM NaCl	7.4	~100	3.5 [w]	[73]
**YT-WR**	VKV**W**VK**Y**^D^**NG**TKV**R**VKV-NH_2_			~1000	n.r.	
**YT-2W**	VKV**W**VK**Y**^D^**NG**TKV**K**V**W**V-NH_2_			~1000	n.r.	
**ASCP1**	**(KIGAKI)_3_T**^D^PP**G(IKAGIK)_3_**-NH_2_	50 mM borate	9	~50	7–10 [w] 1.7 [h]	[74]

## Data Availability

No new data were created or analyzed in this study.
